# Crossing epigenetic frontiers: the intersection of novel histone modifications and diseases

**DOI:** 10.1038/s41392-024-01918-w

**Published:** 2024-09-16

**Authors:** Weiyi Yao, Xinting Hu, Xin Wang

**Affiliations:** 1 Department of Hematology, Shandong Provincial Hospital Affiliated to Shandong First Medical University, Jinan, Shandong 250021 China; 2 Department of Hematology, Shandong Provincial Hospital, Shandong University, Jinan, Shandong 250021 China; 3 Taishan Scholars Program of Shandong Province, Jinan, Shandong 250021 China

**Keywords:** Drug delivery, Oncogenes, Cancer, Epigenetics analysis, Cancer therapy

## Abstract

Histone post-translational modifications (HPTMs), as one of the core mechanisms of epigenetic regulation, are garnering increasing attention due to their close association with the onset and progression of diseases and their potential as targeted therapeutic agents. Advances in high-throughput molecular tools and the abundance of bioinformatics data have led to the discovery of novel HPTMs which similarly affect gene expression, metabolism, and chromatin structure. Furthermore, a growing body of research has demonstrated that novel histone modifications also play crucial roles in the development and progression of various diseases, including various cancers, cardiovascular diseases, infectious diseases, psychiatric disorders, and reproductive system diseases. This review defines nine novel histone modifications: lactylation, citrullination, crotonylation, succinylation, SUMOylation, propionylation, butyrylation, 2-hydroxyisobutyrylation, and 2-hydroxybutyrylation. It comprehensively introduces the modification processes of these nine novel HPTMs, their roles in transcription, replication, DNA repair and recombination, metabolism, and chromatin structure, as well as their involvement in promoting the occurrence and development of various diseases and their clinical applications as therapeutic targets and potential biomarkers. Moreover, this review provides a detailed overview of novel HPTM inhibitors targeting various targets and their emerging strategies in the treatment of multiple diseases while offering insights into their future development prospects and challenges. Additionally, we briefly introduce novel epigenetic research techniques and their applications in the field of novel HPTM research.

## Introduction

Histones, as the fundamental building blocks of chromatin, have their functions and characteristics regulated through an intricate and complex array of HPTMs.^1^ These modifications work in concert to determine the spatial structure of chromatin and profoundly influence the expression and interpretation of genetic information.^2^ The diverse changes brought about by HPTMs offer an additional layer of control over gene expression, enabling cells to respond flexibly to various external stimuli. This adaptability ensures the normal functioning of vital processes while also allowing for rapid reactions to environmental changes.^3–5^ Proteins undergo various HPTMs that are crucial for chromatin regulation and function, affecting gene expression, chromatin structure, and cellular responses to stimuli. While acetylation (ac) and methylation (ma) are well-studied HPTMs, there has been a recent realization that the functions of many HPTMs remain largely unknown. Newly discovered HPTMs include a range of lysine ac such as propionylation (pr), butyrylation (bu), crotonylation (cr), hydroxyisobutyrylation (hib), succinylation (succ), and ma.^6–9^ Many of these ac sites coincide with known ac sites, prompting questions regarding the functional similarities between them.^10^ HPTMs are subject to influence by external environmental factors and metabolic status, and they possess the ability to significantly affect the initiation and progression of a range of conditions, including inflammation,^11^ cancers,^12^ cardiovascular diseases,^13^ kidney diseases,^14^ metabolic disorders^15^ and neuropsychiatric diseases.^16^

HPTMs serve as a pivotal link between cellular metabolism and the control of epigenetic mechanisms, emerging as a focal point of study within this domain.^17^ In the realm of cancer treatment, notably, inhibitors that target HPTMs, including ma and ac, have shown efficacy. For example, demethylating agents and histone deacetylase inhibitors can effectively treat acute myeloid leukemia and T-cell lymphoma.^18^ Furthermore, targeting epigenetic regulatory factors represents an effective strategy for reversing drug resistance.^19^ These advantages have prompted researchers to shift their focus towards the emerging field of HPTMs studies in recent years. With breakthroughs in proteomics research using high-resolution mass spectrometry (HRMS), research has identified nine novel HPTMs,^20^ including lactylation (la),^21^ citrullination (cit), cr,^22^ succ,^9^ pr,^6^ bu,^6^ SUMOylation, hib^8^ and 2-hydroxybutyrylation (bhb).^23^ Recent studies have elucidated the novel mechanistic roles, associations with cancers, and potential therapeutic applications of these HPTMs, revealing substantial practical implications.

## The developmental trajectory of Hptms

The concept of epigenetics, initially introduced by Conrad Hal Waddington in the year 1942 as a mechanism elucidating how the genotype begets the phenotype, has experienced substantial development. From Waddington’s designation of epigenetics as the process through which the genotype engenders the phenotype,^24^ to Nanney’s ^25^focus on the regulatory systems governing gene expression, and further to Riggs,^26^ Holliday,^27,28^ Martienssen, and Russo emphasizing heritable gene function changes that cannot be explained by alterations in the DNA sequence. Bird ^29^construed epigenetics as the adaptive alterations of chromosomal structures, while Greally and Lappalainen posited that it involves gene regulators that endow cells with the capacity to memorialize past events. Lastly, Nicoglou highlighted the stability exerted by intracellular factors on the potentialities of the genome. These definitions collectively mirror the richness and complexity inherent in the domain of epigenetics.^30^ Waddington’s concept of the “epigenetic landscape,” introduced in 1957, emphasizes that cellular differentiation could be regulated by alterations in the epigenetic landscape rather than by changes in the genes themselves.^24,31,32^ Subsequent advancements in epigenetics have resulted in breakthroughs across various aspects: In 1964, the first description of histone modifications closely linked to the regulation of RNA synthesis;^33^ the chromatin nucleosome organization model proposed in 1974, which detailed the basic unit of chromatin;^34^ the discovery of DNA modifications in 1975, particularly 5-methylcytosine, demonstrating its relevance to gene regulation;^28^ and in 1976, Sanger’s identification of circular RNA, along with the first long non-coding RNA (H19) recognized in 1990, blazed new trails for epigenetic regulatory research.^35,36^ In the year 1994, the unveiling of the initial microRNA, lin-4, illuminated the process through which miRNAs orchestrate the regulation of gene expression. This is achieved by their complementary association with specific target mRNAs.^36^ Additionally, the initial discovery in 1996 of histone acetyltransferases (HATs) and histone deacetylases (HDACs) provided insights into the role of protein acetylation in epigenetics.^37,38^ The year 1997 marked a significant advancement in the realm of molecular biology, with X-ray crystallography elucidating the nucleosome core particle’s structure within chromatin. This revelation provided a profound insight into the organizational framework of DNA and the intricacies of its regulatory systems.^39^ Since the turn of the millennium, the field of epigenetics has continued to evolve rapidly. The discovery of SUV39H1 marked the beginning of histone lysine methyltransferase research, with the first histone lysine demethylase LSD1 following closely behind in 2004.^32,40^ In 2006, the FDA approved the first batch of epigenetic drugs for cancer treatment, and in 2012, the first reports of cancer-related histone gene mutations emerged.^41,42^ In 2015, the NIH Roadmap Epigenomics Mapping Consortium released 111 reference human epigenomes.^43^ Over the past five years, a multitude of epigenetic drugs targeting DNA methyltransferases, HDACs, HMTs, and BET proteins have been tested in clinical trials, investigating their efficacy in treating various diseases, both as monotherapies and in combination therapies.^30^ These achievements reflect the multifaceted nature of epigenetics as a concept across different disciplinary contexts, revealing the historical evolution and rich definitions within this field.

Before the early 1990s, it was widely held that histones—compact basic proteins that, in conjunction with DNA, constitute the chromatin framework in the nucleus—simply functioned as structural support for DNA, playing no active role in the regulation of genes.^44^ However, subsequent research has shown that histones play crucial roles in gene expression regulation, DNA damage repair, DNA replication, and recombination. Histones are pivotal mediators in the epigenetic regulatory landscape, influencing inheritable chromatin configurations that transcend the genetic script of DNA. Such a role is indispensable for cellular differentiation, where histones undergo an array of covalent modifications. These alterations span phosphorylation, ubiquitination, acetylation, methylation, along with emergent types of histone modifications such as la, cit, cr, succ, pr, bu, SUMOylation, hib, and bhb. The histone code hypothesis posits that these modifications, occurring singly or in concert on one or several histone tails, operate in a sequential or combinatorial manner, creating a ‘histone code’. This code is interpreted by specific proteins, which then initiate diverse downstream biological outcomes. The resulting histone codes may manifest as transient signals or as more enduring entities, with the latter embodying the true heritable epigenetic code.^45^

**Fig. 1:**
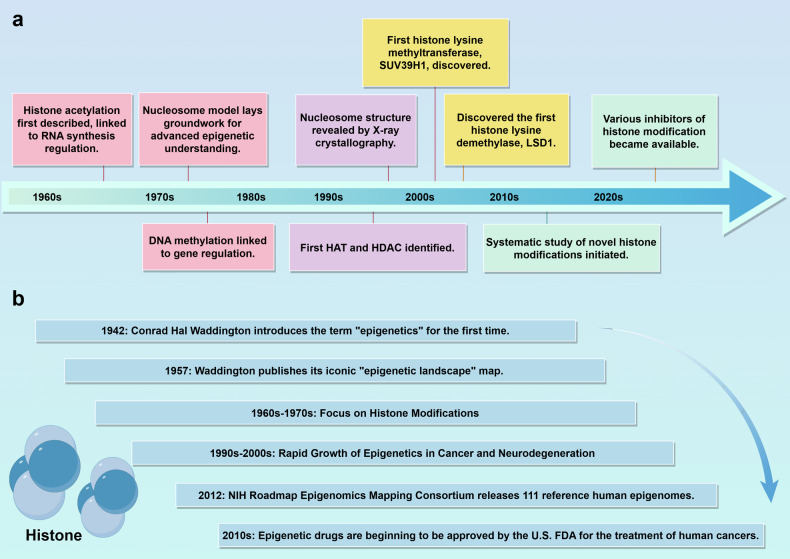
A succinct historical overview of the development of epigenetics and HPTMs. **a** Key discoveries in histone modifications and chromatin biology from the 1960s to 2020s. **b** Chronological overview oflandmark events in the field of epigenetics, including conceptual developments and practical applications in medicine

Histone modifications, as an integral component of epigenetics, were first described in 1964 when histone ac was discovered, playing a crucial role in local chromatin relaxation. This process of ac, by neutralizing the positive charges on lysine residues, diminishes the interaction strength between histones and DNA.^46^ However, it was not until between 1996 and 1998 that, with the development of molecular cloning techniques and protein purification methods, scientists successfully identified and characterized eight different HATs containing acetyltransferase domains. This significant advancement not only enriched our understanding of the regulatory functions of histones but also unveiled the pivotal role of ac in gene expression. Regarding histone ma, its functional role was recognized as early as 1962, but a deeper investigation into its mechanisms became possible only with the identification of HMTs. These HMTs catalyze the ma of histones at specific residues, thereby regulating gene expression and chromatin structure. From 1993 to 2005, scientists gradually identified a range of HMTs responsible for various ma reactions, broadening our comprehension of the complexity and precision of histone functions. As we ventured into the new millennium, a succession of pivotal discoveries concerning novel histone modifications has been made. These have not only enriched our comprehension of cellular biology but have also unveiled new vistas for therapeutic intervention. To begin with, in 2003, Shiio and Eisenman conducted pioneering research on the SUMOylation of histone H4 at lysine 12, marking the commencement of explorations into the mechanisms of histone modification.^47^ Subsequently, in 2007, Chen and colleagues discovered the ubiquitination and proline isomerization modifications of histones, further enriching our understanding of the diversity of histone functions. By 2011, scientists had for the first time identified the succ of histones in mammals, offering a fresh perspective on the regulation of histone modifications.^48^ However, in 2014, Dai’s team, utilizing advanced mass spectrometry techniques and chemical biology methods, identified a novel histone modification known as bu, thereby pioneering a new field in histone modification research.^6^ Subsequently, in 2016, Xie and colleagues identified bhb as a HPTM, further expanding our understanding of the diversity of histone modifications.^23^ By 2017, Tan and others, through a systematic analysis, discovered a new HPTM, cr, highlighting the importance of specific histone modifications in the regulation of gene expression.^7^ Finally, in 2019, a study revealed that an accumulation of lactate could trigger histone la, affecting gene transcription and thereby elucidating the critical role of HPTMs in diseases such as cancer and inflammation.^21^ Fundamentally, this continuum of discoveries underscores the cumulative and forward-moving nature of scientific inquiry. Each stride is scaffolded upon pre-existing knowledge, methodically disclosing the intricate and consequential role played by histone modifications in the orchestration of gene expression and cellular operations. These studies not only provide new theoretical perspectives in biology but also pave the way for future biomedical research **(**Fig. 1**)**.

## Hptms: epigenetics’ key components

### Structure of histones

Epigenetic regulation primarily occurs through four key mechanisms: ma of DNA, modifications of histone post-translationally (often referred to as HPTMs), modulation of the chromatin architecture, and the regulation by noncoding RNAs.^49,50^ Histones, essential components of epigenetic machinations, fulfill two critical nuclear roles: DNA compaction and gene expression regulation. These small, basic proteins are characterized by a globular domain at the C-terminus and a tail at the N-terminus. Within the eukaryotic cell nucleus, they associate with DNA, constructing nucleosomes—the fundamental building blocks of chromatin. Approximately 146 base pairs of DNA coil around a core histone octamer, comprised of two each of histones H2A and H2B, and a tetramer of histones H3 and H4. Additionally, the linker histone H1 fortifies chromatin structure by anchoring both to the nucleosome and the inter-nucleosomal DNA.^51^ Histones are adorned with a variety of HPTMs, primarily occurring on their N-terminal tails.^52^ Histone tails, characterized by their inherent disordered structure and extensive post-translational modifications, are central to transcriptional regulation.^53^ These modifications, which include both activation through positive regulation and suppression via negative mechanisms, endow histones with a dynamic regulatory capacity. Predominantly positively charged due to rich lysine and arginine residues, with about two-thirds of these charges located in the tails, histones modulate interactions with DNA and among themselves, thereby intricately influencing gene activation and repression. In essence, the modifications of histone tails are crucial for the nuanced balance of gene expression control.^54^

In the preceding decade and a half, scientific exploration has evolved markedly. Where initial inquiries were once centered on the HPTMs of histone tails, contemporary studies have shed light on the functional consequences of modifications occurring within the globular domains of histones. The lateral surfaces of the histone octamer, in direct contact with DNA, pose greater challenges in terms of accessibility compared to the more exposed histone tails. Despite this, nucleosomes are dynamic structures; their DNA intermittently unwinds from and rebinds to the lateral surface, consequently providing chromatin-modifying enzymes the opportunity to access and alter these nucleosomal regions.^55^ Numerous HPTMs discerned within the globular domain appear to be situated on the lateral surface or at the junctures between the histones forming the octamer. This observation intimates that alongside the extensively researched histone tail modifications, those within the globular domains are equally integral to the dynamic behavior of nucleosomes. These modifications influence the interactions between DNA and histones, as well as the overall structure and function of chromatin, exemplified by modifications on the lateral surface of the histone octamer,^56^ HPTMs surrounding the symmetry axis,^57^ and modifications at the octamer interfaces.^58^

Furthermore, the codex of histone modifications is characterized by its dynamism and complexity.^50^ For instance, histones can undergo a multitude of modifications on different amino acid residues. Mass spectrometry has unveiled intricate patterns of modifications on histones H2A, H2B, H3, and H4, identifying them as having 13, 12, 21, and 14 potential modification sites, respectively.^59^ These modifications encompass ma, ac, phosphorylation, and ubiquitination, among others. Each site may undergo modification or remain unmodified, thereby amplifying the potentialities and intricacies of these modifications. Notably, lysine residues can not only be methylated or acetylated but, in the case of ma, can exhibit variability with mono-, di-, or trime. Given these combinations and variations, the overall pattern of histone modifications displays a tremendous diversity, constituting a complex regulatory network. Additionally, the code of histone modifications can sometimes change transiently with the cellular milieu, reflecting shifts in the cell’s physiological state and surrounding signals.^29^ Furthermore, research has unveiled that specific “active” histone modification patterns, including H3K4ma2/ma3, H3K79ma2, and ac of H3 and H4, exhibit temporal stability in the promoter regions of genes, persisting even in cells halted in mitosis. This suggests the potential inheritability of such modification patterns.^60,61^ The concept of an inheritable histone code is embraced as an epigenetic code, denoting the transmission of this code from cell to cell across disparate tissues within multicellular entities. Consequently, this influences the expression tableau across the organism’s entirety.^45^

### Enzymes in Hptms

HPTMs are of paramount importance in epigenetic discourse, as they modulate chromatin architecture and gene activity by appending chemical groups to amino acid residues on histone tails. The dynamism of these modifications involves a variety of specialized enzymes, primarily categorized into three groups: “readers,” “writers,” and “erasers“.^62^ “Reader” enzymes recognize and bind to specific chemical modifications, such as ma or ac, on histones, thereby recruiting other proteins or protein complexes to the chromatin and subsequently influencing gene activity. This recognition mechanism is facilitated through specialized domains, enabling precise regulation of transcriptional activity by the “reader” enzymes. “Writer” enzymes are responsible for adding new chemical modifications, such as methyl, acetyl, or phosphate groups, to histones. Enzymes like SETD2, KDM5B, and EP300 play key roles in cellular function and development by catalyzing specific lysine or serine residues and regulating gene expression through these modifications. In contrast, “eraser” enzymes are tasked with removing existing histone modifications. “Erasers” such as demethylases and deacetylases, like HDAC3, affect gene expression and modulate cellular functions by removing modifications, like acetyl groups, from histones.^63,64^The activity of these enzymes is crucial for maintaining the dynamic state of the chromatin and for adaptive modulation of gene expression patterns.^49^

Within the realm of epigenetic regulation, HPTMs involve a myriad of specific enzymes tasked with catalyzing, recognizing, or removing modifications. P300/CBP, as a ubiquitously present acyltransferase, readily facilitates the production of histone pr and bu.^65,66^ The GCN5 and MYST families are specifically dedicated to the targeted inscription of pr.^67^ Within the context of histone ma and succ, the corresponding malonyl-CoA and succinyl-CoA regulate the levels of these modifications under the catalytic influence of acyltransferases. SIRT5 possesses the capacity for demalonylation, while SIRT2 demonstrates similar functionality in yeast.^68^ In the context of bhb, the enzyme P300 adeptly appends β-hydroxybutyryl moieties, whereas SIRT3 is adept at reversing this biochemical process.^69^ Additionally, class I and III HDACs harbor the capacity to excise β-hydroxybutyrate moieties. The regulation of histone glutarylation is managed by GCN5 and SIRT7, with glutaryl-CoA being a determinant in the modulation of glutarylation’s prevalence and localization.^58^ Furthermore, the recognition and catalysis of cr involve the YEATS and DPF domains, as well as CDYL reader structures, with P300 considered to be the sole known octanoyltransferase.^70^ In conclusion, the deacylation of crotonylated histone peptides and proteins is predominantly executed by members of the SIRT family, including SIRT1, SIRT2, and SIRT3. Conversely, the overexpression of HDACs can diminish the concentration of cr marks.^71,72^ The activity of these enzymes is integral to the nuanced regulation of gene expression and profoundly influences cellular metabolism and development, occupying a central position in the context of cellular physiology and pathology.

### The role of dysregulated novel HPTMs in disease pathophysiology

#### Histone lactylation

Lactate is a common byproduct of glycolysis, predominantly converted from pyruvate through the action of lactate dehydrogenase (LDH).^73^ Despite traditionally being considered a metabolic waste product, lactate exhibits anomalous glycolytic behavior in tumor cells, characterized by overproduction even in the presence of ample oxygen—a phenomenon first identified by Otto Warburg, known as the “Warburg effect“.^74^ In recent years, lactate’s role in regulating various intracellular and extracellular dynamic processes has been increasingly recognized, encompassing gene expression, metabolic dynamics, the tumor microenvironment (TME), as well as the activation or inhibition of immune cells.^75,76^

Zhang and colleagues’ research has uncovered histone lysine la as a novel epigenetic modification, which directly originates from exogenous or endogenous lactate.^21^ By employing carbon-13 labeled lactate and glucose, combined with mass spectrometry analysis, the study confirmed that lactate can be converted into lactyl groups on histones. This discovery not only validates the existence of histone la but also provides a new perspective on the intracellular functions of lactate. Further investigation revealed that the dynamics of la and ac differ, with these modifications being regulated differently by glucose metabolism.^77^ The research team also explored the relationship between lactate production and the levels of histone la, finding that glycolytic inhibitors, which reduce lactate generation, decrease the levels of histone la, whereas mitochondrial inhibitors or hypoxic conditions that increase lactate production led to an increase in histone la. Moreover, the study highlighted that various histone lysine ac, such as bu, pr, and hib, are associated with changes in the rate of glycolysis.^7,69^ Recent studies indicate that controlling the concentration of acetyl coenzyme A (the substrate for acetyltransferases) can effectively regulate the levels of histone ac.^78^ Overall, the research conducted by Zhang and colleagues illuminates the significant role of histone la in cellular metabolism and epigenetic regulation, particularly under hypoxic conditions, where an increase in lactate independently of the acetyl coenzyme A regulatory pathway directly influences the elevation of histone la levels. These findings pave the way for future research into how lactate can affect cellular functions through epigenetic mechanisms.

Lactate is a known energy source for cancer cells, which shuttle lactate to adjacent cancer cells, the surrounding stroma, and vascular endothelial cells, thereby inducing metabolic reprogramming.^79^ Lactate not only contributes to the promotion of tumor-associated inflammation but also serves as a signaling molecule that can stimulate angiogenesis within the tumor milieu. However, the non-metabolic effects of lactate at high concentrations remain unclear.^80^ In 2019, studies showed lactate accumulation triggers histone la, influencing gene transcription, and suggesting its significant role in conditions like cancer and inflammation.^21^ Additionally, many HPTMs are not enzymatically regulated but are merely chemical by-products. Additionally, metabolic intermediates serve not only as sources of chemical substances but also regulate gene expression by influencing HPTMs. Evidence of “writers” and “erasers” confirms that their regulation is at least partially enzyme-mediated, thereby making HPTMs both targetable and directly exploitable.^81,82^ Recent research connects histone la to cancer progression, emphasizing lysine la’s importance in altering cancer cell metabolism.^83^ Disruption of histone la unbalances gene transcription, leading to cancer and various diseases. Recent research connects histone la to cancer progression, emphasizing lysine la’s importance in altering cancer cell metabolism.^83^ Disruption of histone la unbalances gene transcription, leading to cancer and various diseases. Moreover, existing studies indicate a strong link between histone la and cancer prognosis.

#### Histone citrullination

Histone citrullination is a HPTM catalyzed by the peptidylarginine deiminase (PAD) family, a process contingent upon elevated calcium concentrations. Under pathological conditions, PAD enzymes have the ability to citrullinate various structural proteins.^84^ Studies suggest that histone cit is implicated in autoimmune conditions, exemplified by rheumatoid arthritis (RA), wherein the presence of anti-citrullinated protein antibodies is a distinct marker for the disease. They can be utilized for early detection, reflect disease outcomes, and serve as valuable diagnostic and prognostic tools for RA.^85,86^ Considering the involvement of PADs in both normal physiological functions and disease processes, research and application of PAD inhibitors have been pursued. Numerous PAD inhibitors are utilized for the treatment of PAD-associated diseases in the skin, joints, colon, and immune system.^87–90^

PADs are regarded as transcription-regulating proteins that influence gene expression, the precise biological functions of citrullination remain unclear.^91^ Citrullinated histones account for about 10% of all histone molecules in HL-60 granulocytes, underscoring the significance of this HPTMs in numerous nucleus-associated processes.^92^ Cit plays a vital role in embryonic development; studies have demonstrated that the use of a specific PAD1 inhibitor significantly reduces cit of H4R3 and H3R2/8/17 in embryonic cells, coinciding with a developmental arrest at the four-cell stage.^93^ Moreover, in a zebrafish tissue injury model, PAD2-mediated H4 cit is essential for effective regeneration, posited as a potential intermediary between early calcium signaling and subsequent wound healing.^94^ The linker histone H1.0, when citrullinated, accumulates in aging cells and is involved in heterochromatinization and the aging process.^95^ PAD2 modulates the expression of genes related to lactation through histone cit.^96,97^ Another function of citrullinated histones is to modulate the formation of neutrophil extracellular traps (NETs).^98,99^ In innate immunity, neutrophils are the first responders to bacterial infection, combating various pathogens by forming so-called NETs.^98^ The enzyme PAD4-mediated cit of histones plays a pivotal role in the genesis of NETs. Neutrophils deficient in PAD4 fail to produce NETs upon exposure to chemotactic stimuli or bacterial incubation, highlighting the essentiality of PAD4 in the NET-dependent antibacterial response.^100^ Research has also discovered that inhibiting PAD2 can reduce the formation of NETs and the production of inflammatory cytokines in sepsis, suggesting that PAD2 may play an important role in regulating both NET formation and inflammatory responses.^101^ In cases of COVID-19, an increase in the quantity of NET remnants has been observed in patients’ serum, indicating that healthy neutrophils, upon exposure to serum samples from COVID-19 patients, undergo NETosis more frequently.^102^ Histone cit contributes significantly to embryonic development, reproductive functions, chromatin expression, dissolution, pluripotency, and the formation of NETs.

Histone cit by PAD enzymes is intricately linked to cancer progression, impacting tumor development, gene regulation, cell differentiation and cell death, and plays a key role in chromatin activity modulation. PAD inhibitors have demonstrated immense potential in the field of cancer therapy, with PAD4 inhibitors in particular being applied to prevent tumor metastasis and associated thrombosis in cancer patients.^103^ Histone cit by PAD is increasingly recognized as a diagnostic marker and treatment target in cancer research. Investigations have revealed that in a spectrum of malignancies—among them non-small cell lung cancer (NSCLC), gastric cancer, hepatitis B virus-associated hepatocellular carcinoma (HCC), and various malignant hematological disorders—PAD-mediated cit of histones is notably elevated.^104^

NETs are observed in a variety of human cancer types, recognized as contributors to cancer progression.^105,106^ PAD4, an enzyme abundantly found in various cancers and neutrophils, plays a critical role in the formation of NETs.^95^ Additionally, the development of NETs driven by histone cit is closely associated with tumor proliferation and spread, involving processes such as ECM reorganization, surgical stress response, hypoxic conditions, alterations in fatty acids and cellular interactions through physical binding.^104^ Corroborating this perspective, Demers et al. utilized PAD4-deficient mice, which intrinsically exhibit impaired neutrophil chromatin decondensation and diminished NETs generation capabilities. Their findings insinuated that when neutrophils initiate NETosis, it inadvertently promotes tumor proliferation. Thus, the study suggested that tumors, or the environment they exist in, trigger neutrophils to undergo NETosis, leading to a build-up of NETs within the tumor itself, which in turn promotes tumor proliferation.^107^ In summary, histone cit contributes to cancer progression and dissemination through NET production.

#### Histone crotonylation

Histone cr was first identified by Tan and colleagues through the analysis of MS data. Utilizing the PTMap software to pinpoint post-translational modification sites, they uncovered 28 potential butyrylated histone markers.^7,108^ Tan and colleagues have discovered that novel histone modifications mark TSS of active genes with specificity for cr, predominantly located at active promoters, which are critical to the regulation of gene expression. This finding underscores the significant role of histone cr in modulating chromatin structure and function.^7,109^ Histone cr, much like ac, invariably takes place on lysine residues and is dynamically governed by the enzymatic actions of crotonyltransferases and decrotonylases.^110^ Cr is differentiated from ac within the histone modification landscape by its distinct structural features, including a four-carbon planar chain and an alkenyl C = C double bond, which confer upon it unique functional properties.^70^ In the mechanistic dance of cr, crotonyltransferases orchestrate the donation of crotonyl groups from crotonyl-CoA to specific amino acid residues on histones, while decrotonylases play their part in excising these crotonyl moieties, ensuring a dynamic equilibrium. HATs also play a regulatory role in the cr of histones, while a class of HDAC1, 2, 3, 8 operate as decrotonylases. Furthermore, studies have found that the concentration of crotonyl-CoA is a limiting factor in histone cr.^111,112^ Cr is a highly dynamic modification, capable of either activating or repressing transcription. Cr exerts a more significant impact on the regulation of cell cycle and metabolism compared to ac, with its influence being dependent on particular genes or environmental conditions. In both human somatic cells and mouse male germ cells, cr is observed at the promoter regions of actively transcribed genes or at enhancers, where it plays a pivotal role in the modulation of gene transcription.^7,111^ Histone cr is a critical element in orchestrating a multitude of biological pathways, including the response to acute renal damage, the maturation of sperm cells, the preservation of chromosomal end structures, the dormancy of the human immunodeficiency virus, and the advancement of oncological diseases.^14,113–117^

#### Histone succinylation

The phenomenon of histone succ first emerged in scientific literature with its identification in mammals in 2011.^48^ Histone succ was first identified in the activity of homoserine succinyltransferase and subsequently confirmed as a natural and novel post-translational modification through methods such as Western blot analysis, isotopic labeling, tandem MS/MS, and co-elution experiments using high-performance liquid chromatography (HPLC).^118,119^ This entity has been detected across diverse biological systems, ranging from the bacterium Escherichia coli, unicellular yeast organisms, the protozoan Toxoplasma gondii, to the cultured human cervical cancer cells known as HeLa, and extending to the hepatic tissues of mice.^9,120^ Discoveries across various cell types indicate a high degree of evolutionary conservation among species. Many of the identified succ sites overlap with sites of other HPTMs, such as ac, ma, and hib, suggesting intricate interactions between histone modifications. The identification of lysine succ has also led to the discovery of other similar modifications, such as pr and bu.^121^ Succ can exert a direct influence on the organization of chromatin by chemically altering histones or by modulating interactions within the nucleus. This process can induce spatial conformational changes within the chromatin structure that are more substantial than those caused by ma or ac.^9,69^ The presence of succinyl groups on lysine residues can attenuate the interaction between DNA and histones, thereby destabilizing nucleosomes and chromosomal integrity. This weakening of associations permits DNA to disengage more readily from its protein constraints, enhancing the accessibility of transcription factors to DNA sequences. The consequence is an upregulation in the transcriptional activity of genes.^122^

Numerous studies support the notion that enzymes can catalyze the succ of histones. For instance, p300 can induce succ on synthetic histone tail peptides in vitro.^121,123,124^ Atsushi Yokoyama and colleagues have provided compelling evidence that the succ of nuclear histones is an enzymatically driven process.^125^ Moreover, studies spearheaded by Wang et al. have illuminated that the enzyme lysine acetyltransferase 2 A (KAT2A), also recognized as GCN5, possesses a preferential binding affinity for succinyl-CoA over acetyl-CoA.^126^ The α-ketoglutarate dehydrogenase complex, comprising three constituent components, facilitates the production of succinyl-CoA within the nucleus, thus supplying the required substrate for KAT2A to catalyze histone succ. Contrastingly, a segment of research posits that enzymatic action might not be pivotal in the succ of histones. Research by Simithy et al. has found that the succ of histones H3 and H4 is primarily mediated by non-enzymatic actions under conditions involving a variety of HATs and non-enzymatic circumstances.^127,128^ Elevating the concentration of succinyl-CoA can increase the abundance of nuclear succ. Acidic acyl modifications, including ma, succ, and glutarylation, are more amenable to non-enzymatic catalysis in the absence of enzymes.

Histone succ orchestrated by KAT2A might be a key player in disease pathogenesis, as seen in the emergence of human pancreatic ductal adenocarcinoma (PDAC) and in the context of Hepatitis B virus infection. KAT2A’s significant influence on gene regulation and cell proliferation is exemplified by the observation that, within PDAC samples, the succ at histone H3 lysine 79 augments the levels of 14-3-3ζ and β-catenin. This modification is correlated with the modulation of cellular glycolytic pathways and the enhancement of cellular migration and invasiveness.^129^ Research shows histone succ affects various biological processes, including protease function and gene control, influencing diseases like cancer, cardiac, hepatic disorders and neurodegeneration.^130^ Recent studies increasingly show histone succ’s key role in tumor growth and progression, suggesting new paths for cancer treatment.

#### Histone SUMOylation

Histone SUMOylation, the bonding of SUMO proteins to histones discovered by Shiio and Eisenman in 2003. They investigated the SUMOylation of histone H4 at lysine 12 and discussed the potential role of this modification in gene repression.^47^

Although histone ma, ac, and ubiquitination have been extensively studied, research into histone SUMOylation has been challenging due to its extremely low abundance in cells and the lack of specific antibodies.^47,131^ Histone SUMOylation occurs across a variety of organisms, including yeast, protozoa, and plants, and is involved in the regulation of transcription, centromere assembly, chromatin structure modulation, and double-strand break repair, among other functions. A specific lysine residue, K12, in histone H4 emerges as a major recurrent site of SUMOylation, which is typically associated with the suppression of gene transcription. Biophysical studies have suggested that H4K12succ is incompatible with the compact chromatin structures linked to transcriptional silencing. Further biochemical investigations have revealed that H4K12succ enhances the activity of the specific histone demethylase LSD1 within nucleosomes for H3K4ma2.^132^ Evidence has shown that H4K12succ directly inhibits transcription mediated by RNA Polymerase II on chromatin templates and engages in direct negative crosstalk with p300-mediated histone ac and Set1/COMPASS-mediated histone ma, modifications typically associated with active gene transcription.^133^

#### Histone propionylation and histone butyrylation

In 2007, Chen et al. were the first to discover bu and pr of histones.^6^ These HPTMs display a considerable level of evolutionary conservation across eukaryotic species, as evidenced by their presence in yeast cells, murine hepatic tissue, and the U937 human leukemia cell line.^65,134,135^ Propionyl-CoA and butyryl-CoA act as substrates, donating the propionyl and butyryl moieties respectively, which are indispensable for the ac reactions leading to protein pr and bu.^69^ Histone lysine pr and bu are chemically similar to ac, with the addition of extra carbon atoms, suggesting that they may serve as analogs to ma.^6,136^ These HPTMs are proposed to be catalyzed in vitro by HATs, although evidence of their occurrence in vivo remains unclear.^137^ The activity of enzymes that modify histones, such as HATs, is regulated by the concentration of intracellular metabolites, such as acetyl coenzyme A, which act as cofactors for the enzymes, thereby linking the chromatin state to cellular metabolism.^138,139^ Propionyl CoA and butyryl CoA, related to acylation, serve as intermediates in fatty acid and amino acid metabolism. Studies identifying and mapping H3K14pr in vivo indicate its enrichment at active TSS and promoters within the mouse liver, correlating it with transcriptional activity across various metabolic states.^135^ While H3K14pr attracts specific binding partners, the role of H3K14bu remains less understood. It may prevent the recruitment of certain complexes, or possibly attract other binding proteins not identified in this study. Fluctuations in global H3K14pr levels suggest that histone pr plays a role in metabolic signaling and could be implicated in metabolic diseases, although the specific genomic changes warrant further investigation.^140^ Researchers suggest a possible association of histone bu and pr with conditions such as systemic lupus erythematosus, alcohol dependency, virus-induced cancer development and aberrant gene expression in oncological diseases.^141^

Researchers have identified specific form of histone bu termed lysine isobutyrylation. Notably, this isobutyrylation stems from the catabolism of valine and the oxidation of branched-chain fatty acids, pointing to its extensive regulatory roles in epigenetics and cellular physiology.^142^ Currently, the link between histone isobutyrylation and tumors is still unclear, with future research expected to explore this further.

#### Histone 2-hydroxyisobutyrylation

The Dai team identified a novel HPTMs, hib, through MS and then verified it through chemical and biochemical methods. The modification in question has been validated at 63 lysine hib sites within both human and murine histones. Investigations have determined that the histone hib mark is highly preserved across species and extensively dispersed, possesses elevated stoichiometry, and prompts notable alterations in structural configuration. These discoveries underscore the fundamental role of the histone hib mark in the governance of chromatin functionality.^8^

Lysine hib, ac, and cr are positioned on separate residues within histones, each exhibiting distinct functional attributes divergent from those of lysine ac.^7^ Research has pinpointed 63 instances of histone hib marks, a number that eclipses the identified histone lysine ac sites. Unlike the predominantly N-terminal tail localization of known cr and ac modifications, hib marks are discernible within both the N-terminal regions and the core globular domains of histones. Structurally, hib modifications diverge notably from lysine modifications such as ma, ac, or bu. Hib modifications do more than merely neutralize the positive charge of lysine; they also significantly expand its radius, indicating profound structural and functional implications.^8^ Essentially, the hib modification is characterized by the addition of a hydroxyl group to the lysine residue. This confers upon the modified lysine the capacity to engage in hydrogen bonding with other molecular entities. Such a characteristic is of noteworthy importance, as it can substantially influence the modulation of protein functionality.^143^ The genesis of hib is postulated to occur through an enzymatic process that utilizes a high-energy donor molecule, possibly sourced from 2-hydroxyisobutyrate, such as HibCoA, as a cofactor. This suggests a profound interconnection between cellular metabolism and epigenetic frameworks. Consequently, the hib pathway might provide a means for cells to adapt their epigenetic landscapes in accordance to fluctuations in the cellular concentration of HibCoA. The extent to which enzymes known to regulate lysine ac are involved in modulating hib remains an area of uncertainty. Initial in vitro assessments indicate that HDACs 1-3 have the potential to detach the 2-hydroxyisobutyryl moiety from its lysine counterpart under controlled experimental conditions; however, the validity of this activity within a cellular context awaits further verification. As a novel regulatory factor in genetic regulation, hib is closely associated with the metabolic state of the cell and plays a role in epigenetic regulation.^138,144^ This paves the way for future research into the biological functions and regulatory mechanisms of hib.

#### Histone 2-hydroxybutyrylation

**Fig. 2:**
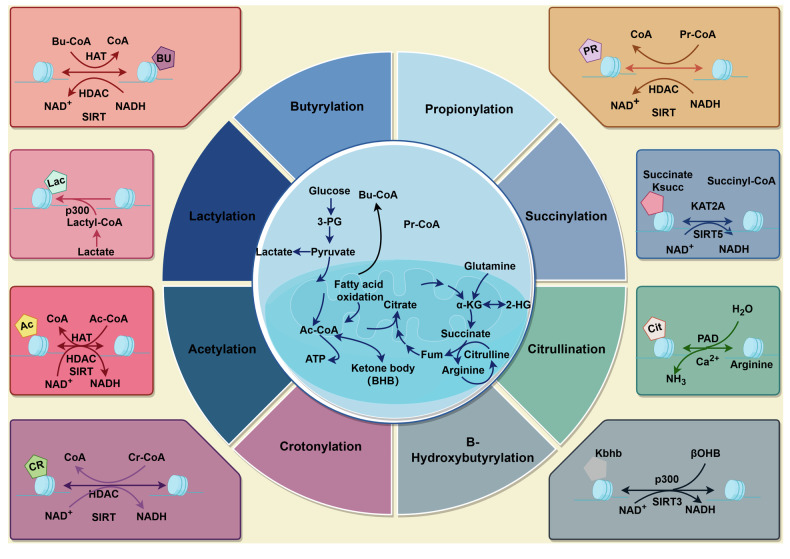
HPTMs and metabolism correlation diagram. Lysine acylation is a complex process interconnected with major metabolic pathways. Glucose, fatty acids, and amino acids serve as primary metabolic resources, producing a wealth of intermediate products within cells, such as lactate, succinyl-CoA, acetyl-CoA, and β-hydroxybutyrate. These intermediates supply acyl groups essential for the covalent modification of proteins. Notably, metabolites like cr, bu, and pr, as well as hib, predominantly arise from fatty acid oxidation and amino acid metabolism. Conversely, metabolites such as la, bhb, and succ primarily originate from glucose metabohighlighting a diverse metabolic sourcing for lysine acylation

Initially delineated by Xie and colleagues in the year 2016, the histone modification termed bhb is dynamically modulated by the intracellular concentration of β-hydroxybutyrate. Despite this established correlation, the exact biochemical pathway through which β-hydroxybutyrate donates β-hydroxybutyryl groups to histones has yet to be fully expounded.^23,145^ Investigations employing RNA sequencing techniques in conjunction with analyses utilizing the KEGG have demonstrated a robust correlation between the bhb of H3K9 and the upregulation of gene expression. When the concentration of β-hydroxybutyrate is elevated, bhb is highly expressed on histones. As an energy source during fasting states for the heart and brain, and in ketogenic diets, β-hydroxybutyrate plays a critical role. β-hydroxybutyryl-CoA, acting as a specific cofactor, catalyzes the formation of bhb in a concentration-dependent manner.^69^ bhb is predominantly enriched at the promoters of active genes and is associated with genes that are upregulated in metabolic pathways responsive to starvation.^23^ β-Hydroxybutyrate, a naturally occurring ketone body, is pivotal in the initiation and advancement of neurological disorders, underscoring its significant impact on neurobiological health. Through comprehensive research, scientists have discovered that the biological process of bhb can provide neuroprotection, effectively mitigating the toxic damage faced by neurons. Furthermore, this process also helps in preventing the degenerative changes in dopaminergic neurons among patients with AD and PD.^146,147^ And p53, recognized as a pivotal tumor suppressor, undergoes modification through bhb at lysines 120, 319 and 370. Under conditions of starvation, researchers noted an elevation in β-hydroxybutyrate serum concentrations in mice, accompanied by a rise in p53 bhb. This process hampers the ac of p53, leading to the cessation of cellular proliferation and a reduction in programmed cell death.^148^ This suggests to us the role of bhb in ketone metabolism and tumor management (Fig. 2) **(**Table 1).

**Table 1:** The overview of novel histone modifications

Histone Modification	Writer(s)	Eraser(s)	Reader(s)	Metabolite Sources	Discovery Year	Function Summary	Reference
Crotonylation (Kcr)	P300/CBP; GNATs; MYSTs	HDAC3; SIRT1-3	YEATS domain; Af9/Taf14; ENL	Crotonyl-CoA; crotonate	2017	Activate transcription;^153^ Regulate spermatogenesis;^7^ Regulate DNA damage response;^476^ Ensures accurate spindle positioning;^477^ Protect renal function;^14^ Ameliorate depression;^16^ Reactivate latent HIV;^116^ Facilitate telomere maintenance and differentiation of stem cells;^115^ Participates in the development of colon cancer;^213^ Impacts the prognosis of prostate cancer;^225^ Regulates the process of spermatogenesis;^114^ Exacerbates major depressive disorder;^275–277^ Plays a role in the occurrence of acute kidney injury and Immunoglobulin A nephropathy.^14,296^	^7,14,16,114–116,153,213,225,275–277,296,476,477^
Lactylation (Kla)	P300	HDAC1-3, SIRT1-3	NA	Lactyl-CoA, L-lactate	2019	Activate transcription^478^; Facilitate cell reprogramming^478^; Promote M1-M2 polarization^21^^,^^479^; Foster lung fibrosis^294^; Accelerate ocular melanoma development^205,206^; Influences on the growth of bladder cancer^207^; Enhancement of glycolysis in acute myeloid leukemia cells^208^; Affects the prognosis of colon^210^、 breast cancer^210^ and clear cell renal cell carcinoma^209^; Reflects the severity of critical illness and the presence of infection^246^; Modulates the transcription and latency of the HIV virus^116^; Contributes to the maintenance of myocardial contractile function^480^; Accelerates the progression of psoriasis.^304^	^21^^,^^116^^,205–210^^,^^246,294,304^^,478–480^
Succinylation (Ksucc)	GNATs(GCN5);HAT1; P300/CBP	SIRT5; SIRT7	YEATS domain(SGAS1)	Succinyl-CoA; Succinate	2011	Activate transcription^481^; Impair mitochondria respiration, mitophagy and metabolic flexibility^482^; Deter neuro filaments aggression in AD^483^; Exacerbate hypertrophic cardiomyopathy and ischemia-perfusion injury^484^; Contributes to the progression of hepatocellular carcinoma, pancreatic cancer, and cholangiocarcinoma.^217^	^217,481–484^
Propionylation (Kpr)	P300/CBP; GNATs; MYSTs	SIRT1-3	Bromodomain; YEATS domain	Propionyl-CoA; Propionate	2007	Activate transcription^135^; Facilitate protein aggregation in neurodegenerative diseases^485^;	^135,485^
Butyrylation (Kbu)	P300/CBP; MYSTs	SIRT1-3	Bromodomain(BRD4,BPTF); CECR 2; AF1	Butyryl-CoA; Butyrate	2014	Activate transcription^486^; Promote spermatogenesis^467^;	^467,486^
2-hydroxyisobutyrylation (Khib)	P300; MYSTs	HDAC1-3; SIRT1-3	NA	2-hydroxyisobutyryl-CoA	2016	Activate transcription^8^; Promote spermatogenesis^8^; Facilitates the onset of pancreatic cancer and oral squamous cell carcinoma^228,229^	^8,228,229^
2-hydroxybutyrylation (Khbb)	P300	SIRT3; HDAC1-3	NA	β-hydroxybutyryl-CoA	2016	Activate transcription^23^; Promote memory development of CD8+Tmem cells^487^; Antagonize glomerulosclerosis induced by diabetes^488^; Alleviate depressive behaviors^290^; Exacerbates major depressive disorder.^275–277^	^23,275–277,290^^,^^487^^,488^

## Biological functions Of Hptms

### HPTMs and genome function

#### HPTMs in transcription

During transcription, HPTMs of histones play a crucial role. These modifications often occur at specific genomic locations, notably enriching at genes, where their presence is associated with transcriptional activity, either positive or negative.^149^ HPTMs is frequently linked to transcriptional engagement, being enriched at active promoters, enhancers, and other regions of chromatin that are readily accessible. Furthermore, it has been demonstrated that HPTMs directly augments the rate of transcription within in vitro settings.^150,151^ Additionally, histone lysine residues are subject to modifications by various long-chain acyl groups. However, these types of modifications are generally observed with much lower prevalence compared to ac.^152^ A quintessential example is histone crotonylation,^7^ which was initially identified as a positive regulator of transcription.^153^ It is of particular interest to note that cr, while typically implicated in gene activation, has also been associated with the repression of gene expression within yeast organisms.^154^ Early studies indicated that the global levels of HPTMs are not related to transcriptional activity.^155,156^ The consequence of HPTMs is highly contingent upon the specific site of occurrence and it is not likely to exert a direct influence on nucleosome architecture.^149^ Although numerous HPTMs have been identified in association with transcription, direct evidence supporting their causal role in transcriptional regulation remains elusive.^157^ Herein, we contemplate the role of specific HPTMs in the regulation of transcription, with particular emphasis on the position of the modified amino acids within the histones.

An array of specific modifications occurs at the histone tails, such as the H3K4ma3, which is concentrated near TSSs and aids in the recruitment of transcriptional machinery. This includes the transcription initiation factor TFIID subunit 4, which facilitates the expression of particular genes and is associated with the maintenance of transcriptional activity in a quiescent state in mammals.^158,159^ Moreover, experiments in fruit flies and African clawed frogs have demonstrated that H3K4ma1 is essential for the memory of an active transcriptional state.^160–162^ Particularly in the context of novel HPTMs, researchers have employed cell-free assays to demonstrate that histone la, akin to acetylation, can directly stimulate gene transcription. Experiments using l-lactyl-coenzyme A instead of acetyl-coenzyme A demonstrated p53-dependent, p300-mediated la of H3 and H4 and the corresponding transcriptional effects. The direct mediation of transcription by histone la was confirmed using recombinant chromatin with lysine-to-arginine mutated core histones. Histone la is not essential for the induction or repression of pro-inflammatory genes but is employed to initiate the expression of homeostatic genes traditionally associated with M2-like macrophages. The aerobic glycolytic switch that occurs during M1 polarization triggers a “lactate timer” that induces M2-like characteristics at a later stage through epigenetic mechanisms, potentially aiding in repairing collateral damage suffered by the host during infection.^21^ Furthermore, the distribution of the epigenetic mark H3K9bhb on histone H3 correlates with alterations in gene expression, capable of reshaping the chromatin landscape and transcriptional responses in brain cells. Histone cr plays a dual regulatory role in gene expression, acting as both a transcriptional activator and repressor, depending on its location and associated genes. Sirt3 was found to reduce the expression levels of Ptk2, Tshz3, and Wapal and decrease the enrichment of histone cr at the promoters of these target genes, indicating that histone cr may serve as a positive regulatory factor for the expression of these genes.^71^ For instance, one study discovered that H3K9cr peaks at pro-growth genes led to gene repression, suggesting that H3K9cr is associated with the transcriptional suppression of pro-growth genes.^154^

In addition to the modifications occurring on histone tails, modifications to core histones exert significant influence on gene expression. Lateral Surface HPTMs can directly affect the binding affinity between histones and DNA, as well as the rate at which DNA unwinds and rewinds.^55,56^ Modifications on the lateral chains of histones can modulate the accessibility of DNA within nucleosomes and promote nucleosome mobility. This particular modification accelerates the rate of local DNA unwinding and induces spontaneous local conformational changes, phenomena colloquially termed as “DNA breathing.” Consequently, this fosters an enhanced affinity for transcription factor binding in vitro experiments.^163,164^ HPTMs around the symmetry axis decrease the overall affinity of DNA for the histone octamer, thereby reducing nucleosome stability.^57,58,124,165^ These core modifications exert their effects through reader or effector proteins.

The direct impact of multiple histone tail HPTMs on nucleosome stability and chromatin architecture is limited, and they typically exert biological effects through the recruitment of binding proteins, or effectors. Conversely, HPTMs that are located within the core domain of the histone octamer are inclined to exert a more direct impact on nucleosome structure and functionality. These intrinsic alterations have the potential to impinge upon chromatin-dependent processes, and this can occur independently of the presence of specific reader proteins. These insights offer a significant perspective on the dynamic modulation of chromatin structure and its impact on the regulation of gene expression, revealing a complex and nuanced interplay between histone modifications and transcriptional regulation. Through the study of these mechanisms, we can gain a more profound understanding of the expression and regulation of information within the cell, as well as unveil potential new strategies for the treatment of diseases, particularly cancer and genetic disorders.

#### HPTMs in recombination

In eukaryotic organisms, meiotic recombination and V(D)J recombination are two critical DNA recombination processes that play a central role in genetic diversity and the development of the immune system. Both processes involve specific HPTMs.

Meiotic recombination frequently takes place at genomic locales known as hotspots, which are characterized by an abundance of open chromatin mark. These epigenetic adornments are intricately arranged by PRDM9—a zinc finger DNA-binding protein with testis-specific expression. PRDM9, in synergy with the lymphoid-specific helicase HELLS, constitutes a vanguard complex. This coalition functions to render the chromatin more accessible, thereby enabling the facilitation of meiotic recombination.^166^ Studies have revealed that H4K8la is closely associated with recombination hotspots, which involve mechanisms that process DSBs, such as SPO11, DMC1, RAD51, and RPA2. Moreover, H4K8la has also been detected at meiosis-specific cohesion sites (marked by RAD21L and REC8) flanking the recombination hotspots.^167^ While PRDM9 plays a significant role in delineating the landscape for meiotic recombination, its presence is not indispensable for the recombination process to occur, as evidenced in the context of rats.^168^ In the majority of vertebrates lacking PRDM9, recombination activity shifts toward other open chromatin structures, such as the promoters of active genes. From a topological perspective, the loop anchors of topologically associating domains (TADs) are enriched with H3K4ma3 and contain multiple PRDM9 binding sites. These features may explain why loop anchors can serve as hotspots for meiotic recombination.^169,170^

The process of V(D)J recombination relies on the functionality of proteins produced by the Recombination Activating Genes, which assemble into a complex consisting of RAG1 and RAG2 subunits. This complex is characterized by its recombinase activity and its ability to bind to the highly conserved recombination signal sequences that border the V, D, and J gene segments. These genomic regions associated with V(D)J recombination are marked by active histone modifications. The interaction of the plant homeodomain within RAG2 with H3K4ma3 triggers a conformational alteration in RAG1. This modification is pivotal in enhancing the catalytic efficiency necessary for recombination.^171^

Overall, H3K4ma3 plays a pivotal role in both types of recombination. In meiotic recombination, it interacts with PRDM9, influencing chromatin accessibility and the selection of recombination sites; in V(D)J recombination, HPTMs is directly involved in the activation and functional deployment of the RAG complex, illustrating its guiding role in immune diversity. These findings highlight the dual function of HPTMs in DNA recombination: modulating transcription and directly impacting the recombination mechanism.

#### HPTMs in DNA repair

HPTMs are critically involved in the cellular mechanisms addressing DNA damage response and repair, particularly when confronting double-strand breaks (DSBs), which represent the most severe form of DNA damage. Genomic integrity is continually challenged by DNA damage, which is a hallmark of cancer.^172^ The propagation of γH2AX coincides with the boundaries of TADs and may be facilitated by the process of loop extrusion mediated by cohesin, aiding in the spread of γH2AX from the site of the double-strand break.^173,174^ γH2AX provides a platform for the recruitment of DNA damage signaling factors, which initiate ubiquitination of H1 and H2A histones mediated by the ubiquitin ligases RNF8 and RNF168, triggering downstream repair processes.^174,175^ The functional engagement of these signaling entities is pivotal in dictating the subsequent choice of DNA repair pathways. Double-strand breaks are predominantly mended through two principal mechanisms: Homologous Recombination (HR) and Non-Homologous End Joining.^176^ HPTMs modulate the balance between the two repair pathways by influencing the binding and activity of 53BP1 and BRCA1. The affinity of different reader domains for cell cycle-regulated and DNA damage-dependent HPTMs collectively and distinctively determines the choice of double-strand break repair pathway. There, they act to inhibit transcription and promote the recruitment of DNA repair factors, thereby facilitating the repair process.^177,178^ Furthermore, research indicates that H3K9 la is significantly enriched in the LUC7L2 promoter, activating LUC7L2 transcription to enhance its expression. LUC7L2 mediates the retention of intron 7 in MLH1, thereby reducing MLH1 expression and inhibiting mismatch repair, ultimately leading to TMZ resistance in glioblastoma multiforme (0GBM).^179^ The orchestration of DNA repair and transcriptional processes is further regulated by the intricate interplay of HPTMs. These HPTMs reveal how chromatin states influence a cell’s capacity to respond to and efficiently repair DNA damage by affecting chromatin structure and function during the DNA damage response and repair processes. Histone modifications play a crucial role in maintaining genomic integrity and preventing the development of cancer.

#### HPTMs in replication

**Fig. 3:**
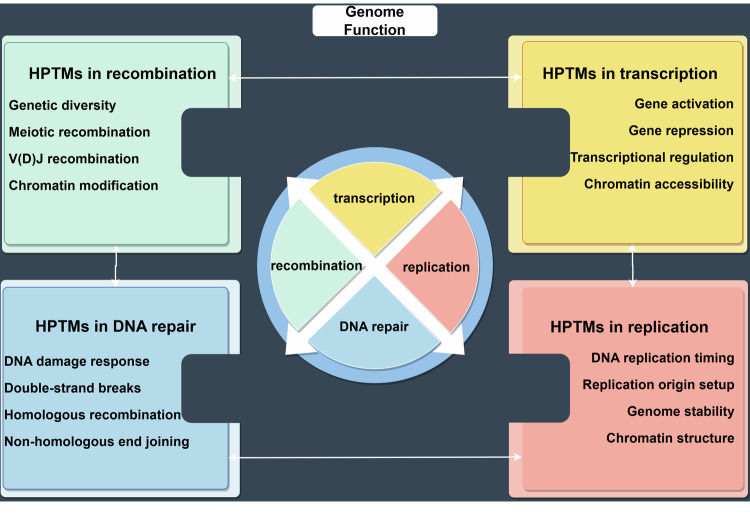
**T**he crucial role of HPTMs in genome function. This figure illustrates the crucial roles of HPTMs in regulating key genomic functions, such as transcription, replication, DNA repair, and recombination

HPTMs play a pivotal role in regulating DNA replication. The modifications, particularly ac and ma on specific histone lysine residues, are essential for the setup and activation of replication origins and influence the overall chromatin structure. These alterations facilitate the assembly of necessary replication complexes, impacting the initiation and efficiency of DNA replication. Additionally, HPTMs are involved in defining the replication timing across the genome, which is crucial for maintaining genome stability and organization. The dynamic relationship between DNA replication and HPTMs suggests that changes in replication timing can alter histone modifications, thereby affecting genomic architecture and compartmentalization within the nucleus. This highlights the significance of a regulated replication process in preserving the integrity and functionality of the genome^180–184^ (Fig. 3).

### HPTMs and cancer metabolism

HPTMs are intimately connected to metabolism. Through metabolic pathways, glucose is broken down into pyruvate and lactate, both of which are associated with specific histone modifications. Lactate is directly linked to histone la, while pyruvate is further converted into acetyl coenzyme A, a substrate for various acylation modifications, including bu. Fatty acids undergo β-oxidation within mitochondria, leading to the production of intermediates like Ac-CoA, which are not only pivotal for energy generation but also fundamental to histone modification processes. For instance, Ac-CoA can directly contribute to histone acetylation. Succinyl coenzyme A and hydroxybutyryl coenzyme A are two other acyl-CoA molecules, which can result in histone succ, bhb, and hib, respectively. The oxidation of long-chain fatty acids also produces various long-chain acyl-CoAs, such as propionyl coenzyme A, which is associated with histone pr. Moreover, amino acids like lysine and tryptophan can be metabolized into their corresponding acyl-CoA derivatives. These acyl-CoA molecules subsequently react with histone lysine residues, leading to modifications such as cr. These processes fundamentally represent the interplay between cellular metabolism and epigenetic modifications, reflecting how cells adjust gene expression and protein function in response to varying metabolic states.

**Fig. 4:**
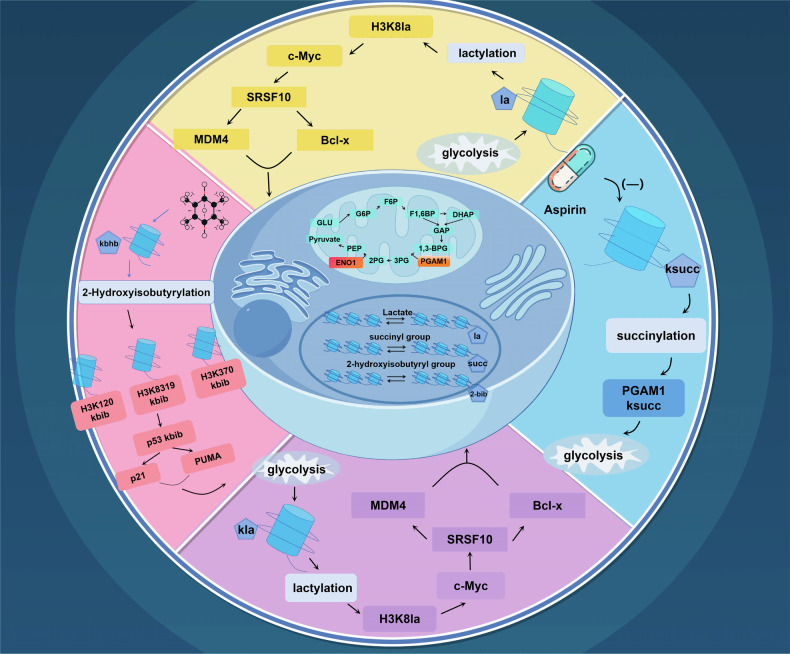
Mechanisms linking novel HPTMs to cancer development through metabolic pathways. The p300 protein possesses key lysine 2-hydroxyisobutyryltransferase activity, playing a crucial role in regulating glycolysis. Aspirin, by specifically hydroxyisobutyrylating at certain sites, inhibits the 2-hydroxyisobutyrylation of the key glycolytic enzyme ENO1, thereby reducing its activity and consequently inhibiting the growth of tumor cells. Additionally, the rate of aerobic glycolysis in tumor cells is increased, accompanied by an increase in histone lactylation, which transcriptionally supports the expression of c-Myc. As a critical transcription factor, c-Myc further upregulates the expression of SRSF10, promoting the selective splicing of MDM4 and Bcl-x in breast cancer cells, thus affecting the growth and survival of cancer cells. On the other hand, aspirin significantly reduces the succ levels of PGAM1 in liver cancer cells, thereby inhibiting glycolysis. Concurrently, 2-hydroxybutyrate induces 2-hydroxyisobutyrylation modifications of p53 at lysine residues 120, 319, and 370. These modifications lead to reduced acetylation levels of p53, subsequently downregulating the expression of downstream genes p21 and PUMA, ultimately resulting in reduced growth and apoptosis of cancer cells

The connection between epigenetics and metabolism is bidirectional, encompassing both research into how HPTMs control the expression of metabolic genes, and investigations into how metabolic pathways influence these newly discovered HPTMs. This bidirectional interaction reveals the complex interplay between epigenetics and metabolic processes, enhancing our understanding of the regulatory mechanisms within organisms. Recent studies reveal that histone modifications, including la, cr, and succ, are intricately linked to cancer metabolism. For instance, histone la bridges cellular metabolism and gene expression, potentially revealing its disease mechanisms. A recent study conducted an analysis of differentially lactylated proteins across various groups, revealing their involvement in a wide range of biological functions. These functions include amino acid and lipoprotein metabolism, as well as the synthesis of ribosomal proteins. This analysis confirmed the close association between histone la and HCC. Furthermore, the investigation confirmed the levels of la on two proteins associated with tumors, namely USP14 and ABCF1, establishing a solid foundation for further exploration of their roles in HCC pathogenesis.^185^ Additionally, the roles of histone cit in NET formation and SUMOylation in cell regulation provide promising directions for cancer therapy development. Furthermore, emerging modifications such as bu and bhb underscore the significance of metabolic pathways in cancer progression, offering new therapeutic possibilities (Fig. 4).

#### Glucose metabolism

Cancer cells primarily rely on glycolysis for energy production. HPTMs, particularly histone la and hib, have the potential to impact cancer therapy by influencing this glycolytic pathway. Some investigations have revealed that p300 possesses 2-hydroxymethylisobutyryltransferase activity, and it modulates cellular glycolysis by amplifying the levels of hib at specific sites in P1. Empirical data indicate that a deficiency in p300 diminishes the activity of enzymes involved in glycolysis, underscoring the potential of modulating hib levels by inhibiting p300 to constrain tumor growth.^186^ The study suggests that in cells affected by neuroendocrine, prostate, or lung cancer, mitochondria are often more fragmented and show a reduced membrane potential, primarily depending on glycolysis for their energy production. Additionally, the interaction between Numb and Parkin promotes mitophagy, crucial for maintaining mitochondrial quality. The Numb/Parkin pathway acts as a critical metabolic regulator and emerges as a potential therapeutic target in oncology.^187^ Aspirin notably decreases overall succ levels in liver cancer cells, including the succ of phosphoglycerate mutase 1 (PGAM1), thereby curtailing the glycolytic process.^188^ For instance, research conducted by Pandkar and their team indicated that decreasing glycolytic activity, especially by inhibiting histone la, significantly reduces the expression of the c-Myc gene, which in turn hinders the advancement of breast cancer.^189^

#### Lipid metabolism

Researchers have found that HPTMs, specifically bhb and bu, are closely associated with ketone body metabolism. In dietary screening studies conducted on spontaneous animal models of CRC, researchers discovered that ketogenic diets possess significant tumor-suppressing effects. This anti-tumor efficacy is replicated through the ketone body β-hydroxybutyrate. β-hydroxybutyrate acts by interacting with the surface receptor Hcar2, subsequently inducing the activation of the transcriptional regulator Hopx. This activation leads to alterations in gene expression patterns, effectively inhibiting the proliferation of colonic crypt cells and significantly suppressing intestinal tumor growth.^190^ Another research indicated that inhibiting the initiation of ketogenesis within the tumor microenvironment is a critical factor in the progression of colorectal cancer. Ketogenic diet has been identified as a key modulator of the tumor microenvironment, capable of diminishing the accumulation of immunosuppressive cells within tumors, enhancing the infiltration of natural killer cells and cytotoxic T cells, and amplifying the anticancer efficacy of PD-1.^191^ Additionally, another study highlighted the positive impact of a ketogenic diet on cancer treatment by restricting glucose and upregulating histone bu, particularly in relation to breast cancer.^192^ One research indicates that β-hydroxybutyrate triggers hib on p53 protein at lysine residues 120, 319, and 370. This cascade of precise molecular regulatory actions leads to a reduction in the ac levels of p53, which in turn permeates through the downstream gene network, significantly suppressing the expression of the genes p21 and PUMA. Ultimately, this series of intracellular signaling events acts in concert to effectively inhibit the proliferation of cancer cells and induces them towards the path of apoptosis, thereby playing a pivotal role in anti-cancer strategies.^148^ However, the possible benefits of a ketogenic diet in cancer treatment, but its definitive effectiveness is still uncertain.^193^ Studies have shown that after long-term treatment, the accumulation of β-hydroxybutyrate and glucose restriction did not significantly affect the levels of bu or ac of histone H3 in cancer cells. Researchers believe that the metabolic plasticity of cancer cells, through limiting glucose and the enrichment of histone modifications, can mitigate or neutralize the effects of long-term metabolic reprogramming. This provides new insights into the controversial mechanism of action of ketogenic diets in clinical trials.^192^

#### Glutamine metabolism

Cancer cells rely extensively on glutamine to fuel their growth, utilizing it in processes like lipid synthesis, the tricarboxylic acid cycle, and for generating amino acids and nucleotides. This ‘addiction’ to glutamine in cancer cells underlines its critical role in the metabolism of tumor cells. Glutamine, absorbed from plasma through different amino acid transporters and transformed into glutamate by mitochondrial glutaminase, plays a pivotal role in glutaminolysis. This process is considered a primary target in the development of cancer therapeutics.^194^ Existing research has outlined the importance of histone ac and glutamine metabolism in cancer, yet the impact of other HPTMs in this context is still not fully understood. Future studies aim to explore their interaction in cancer treatment, potentially identifying new therapy targets. Furthermore, some studies have pinpointed that HPTMs, particularly hib, have a pronounced association with carbohydrate metabolism.^195^

### HPTMs and genome topology

Within the genomes of animals and plants, the chromatin structure is organized into two distinct compartments: A (euchromatin) and B (heterochromatin), each characterized by structural differences.^196^ These compartments are further delineated into topologically associating domains, or TADs. Within the elegant model organism C. elegans, the demethylase of H4K20ma2 stands as one of the key complexes that catalyze the emergence of these TADs.^197^ H3K9 ma plays a crucial role in the compartmentalization of the 3D genome. The deliberate localization of the enzyme that catalyzes the formation of H3K9ma3 to designated sites within human cells, achieved through dCas9-mediated guidance, fosters the tethering of chromatin to HP1α condensates. This strategic recruitment induces a substantial reconfiguration of the chromatin compartments, exemplifying the capacity of targeted histone modifications to alter nuclear architecture.^198–200^ Moreover, H3K27ma3 is equally pivotal in the spatial organization of the genome. It is essential for the formation of chromatin regions known as Polycomb-associated domains, or PADs, through the recruitment of PRC1, yet it is not requisite for their maintenance.^201^ These findings indicate that the genesis of TADs may be delicately orchestrated through the synergistic interactions between DNA and histone modifications. Collectively, HPTMs are deeply entwined with the governance of DNA replication, repair, transcription, and the three-dimensional organization of the genome, serving as pivotal elements in ensuring genomic stability and functionality.

## The role of novel Hptms in diseases

### Cancers

**Fig. 5:**
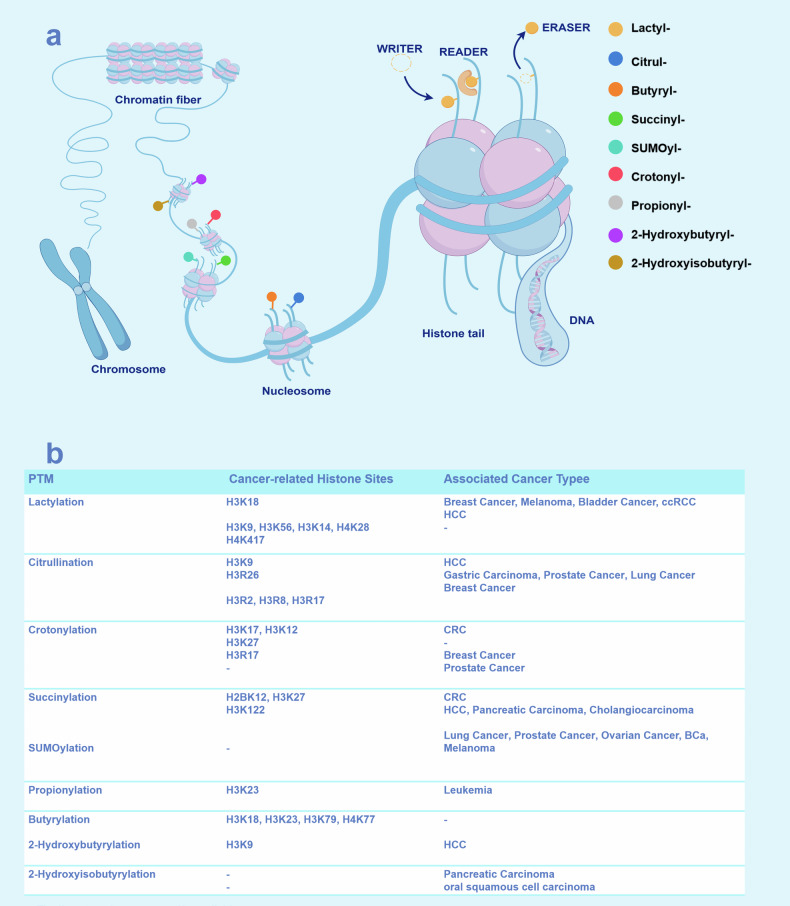
Schematic of HPTMs and the association with cancers. **a** The nine types of histone modifications was added by ‘writers’ and removed by ‘erasers’. These modifications are crucial for the regulation of gene expression. **b** The connections between different modification sites and various types of cancer

**Fig. 6:**
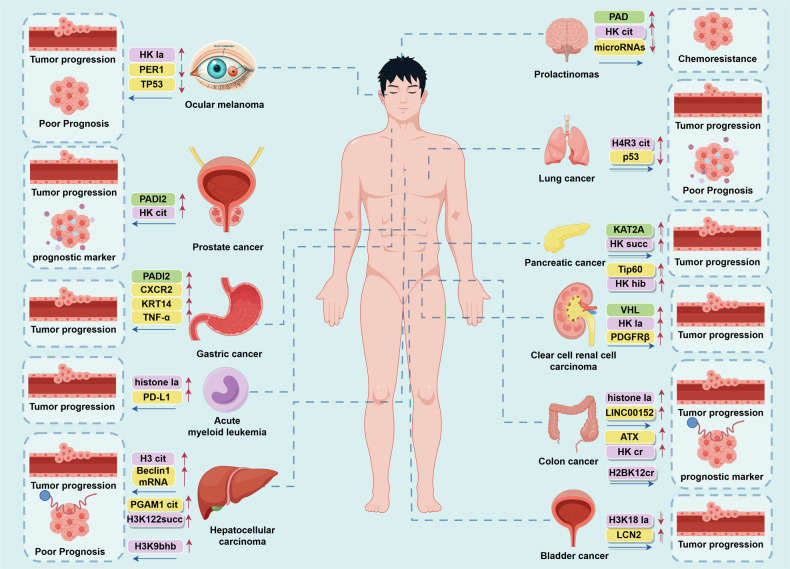
The role of novel HPTMs and related molecular markers in progression and prognosis of various cancer types in the human body. In the context of MI, monocytes first undergo metabolic reprogramming toward glycolysis, becoming dysregulated and committing to increased lactate production. The lactate is then taken up by MCT1 into monocytes, where it accumulates within the cell as a substrate for histone la, leading to histone la-mediated activation and expression of reparative genes including Lrg1, Vegf-a, and IL-10. These effects of monocyte gene induction enable monocytes to exert dual anti-inflammatory and proangiogenic activities, enhancing cardiac repair. Moreover, an increase in calcium ion concentration is the stimulus for PAD4 activation, which brings citrullinated histones, forming NETs. NETs are composed of DNA, histones, and antimicrobial proteins and are released to the extracellular space. These NETs elicit proinflammatory responses, myocardium- and endothelium-damaging effects, and interaction with platelets to elicit TGFβ release that may propagate a myofibroblast-driven fibrotic response. Indeed, NET formation and fibrosis are enhanced in the context of MI, and such responses are absent in mice deficient in PAD4, which have less fibrosis and a favorable outcome in terms of cardiac function

Histone modifications regulate a myriad of physiological mechanisms. Thus, it is unsurprising that their dysregulation is implicated in various complications and diseases. Yet, among the array of conditions bearing the hallmark of epigenetic aberrations, cancer stands as the most thoroughly investigated and distinctly characterized ‘epigenetic disease.’ Feinberg, Ohlsson, and Henikoff have posited that the trajectory of tumorigenesis advances through a tripartite progression: (a) the initial epigenetic disarray within stem/progenitor cells, orchestrated by the aberrant regulation of tumor progenitor genes; (b) the subsequent genetic perturbations affecting tumor suppressor genes and oncogenes; and (c) a phase of compounded genetic and epigenetic volatility, culminating in an accelerated pace of tumor evolution.^202^ DNA ma and various histone modifications play a role in this process, potentially silencing tumor suppressor genes or compromising genomic stability, ultimately resulting in cancer^203,204^ (Fig. 5, 6).

#### Ocular melanoma

Yu et al. discovered that histone 1a can activate the m6A reader protein YTHDF2, which is capable of recognizing m6A modifications on PER1 and TP53 mRNA, thereby promoting their degradation and accelerating the progression of ocular melanoma.^205,206^ Utilizing western blot analysis, researchers uncovered a correlation between increased histone la and adverse outcomes in ocular melanoma patients. A notable feature of many ocular melanomas is the elevated histone la, which might contribute to the tumorigenesis of ocular melanoma.^205^

#### Bladder cancer

a study suggests that the circXRN2-Hippo signaling pathway plays a role in controlling tumor advancement by suppressing H3K18la and regulating the expression of LCN2 in cases of human bladder cancer.^207^

#### Acute myeloid leukemia (AML)

Clinically, researchers have found a positive correlation between the accumulation of lactate in the bone marrow of AML patients and the expression of STAT5 and PD-L1. This correlation is attributed to the overexpression of STAT5 promoting the nuclear translocation of E3BP, thereby activating the promoters of glycolytic genes, which in turn stimulates histone la and induces the transcription of PD-L1. This suggests that patients exhibiting high expression and subsequent production of lactate may benefit more from immunotherapies targeting the PD-1/PD-L1 axis.^208^

Liu and colleagues discovered pr of histone H3 in mammalian cells. Using specific antibodies of pr, they further detected pr in the leukemia cell line U937. The team observed a significant reduction in H3K23 pr within these leukemia cells. Based on these findings, it is conjectured that histone pr might have implications in the pathogenesis of leukemia and potentially serve as a diagnostic marker for therapeutic interventions in leukemia.^65^

#### Clear cell renal cell carcinoma (ccRCC)

Studies have indicated that the expression of histone la is closely associated with cancer prognosis. Particularly in ccRCC, the inactivation of the VHL gene directly increases the expression of histone la, which correlates with poor patient outcomes. Furthermore, the expression of histone la, induced by the inactive state of VHL, further accelerates the development of ccRCC by promoting the transcription of PDGFRβ. In turn, the activation of PDGFRβ also stimulates the transcription of histone la, creating a positive feedback loop that hastens tumor progression. These findings offer significant insights into the molecular mechanisms of ccRCC and may inform the development of therapeutic strategies.^209^

#### Colorectal cancer(CRC)

At the same time, research indicates that the activity of histone la is associated with tumor metastasis and aggressiveness. Wang and colleagues found that lipopolysaccharides from intestinal bacteria can upregulate the expression of LINC00152 in colorectal and breast cancers,^210^ affecting the tumor microenvironment. This upregulation is achieved through an increase in histone la activity induced by lipopolysaccharides and a reduction in the binding affinity of the repressive factor YY1, subsequently promoting LINC00152 expression. Further studies have revealed that overexpression of LINC00152 enhances the migratory and invasive capabilities of cancer cells, highlighting its pivotal role in cancer progression.^211^

The research conducted by Qu and colleagues has revealed that under hypoxic conditions, HIF-300α promotes the development of cancer cells by recruiting p300/CBP to increase Autotaxin(ATX) expression, particularly through the crotonylation of H3 in colon cancer cells, thereby activating ATX. Moreover, the increase of histone crotonylation under normoxic conditions can also initiate the expression of ATX.^212^ In parallel, Liao’s team has established a link between histone crotonylation and DNA damage activity in CRC patients through bioinformatics and Western blot techniques. These findings further highlight the significant roles of histone crotonylation and the cancer therapeutic target ATX in cancer progression.^213^

Hou and his team evaluated the association between histone cr and tumor staging as well as diagnostic outcomes. Their investigation showed a notable increase in H2BK12 cr within the peripheral blood mononuclear cells from patients with CRC. Through ROC curve analysis, it was discerned that utilizing H2BK12cr levels as a diagnostic criterion significantly surpassed conventional carcinoembryonic antigen tests in terms of simplicity and efficacy, thereby highlighting its considerable potential as a tumor biomarker.^214^ Challenging previous views, Liu et al.‘s recent research reveals a new role for histone cr beyond gene activation. Their study suggests that cr at histone H3 lysine 27 (H3K27cr) primarily acts as a suppressor rather than an enhancer of gene transcription. They found that the YEATS domain within the GAS41-SIN3A-HDAC1 complex uniquely recognizes H3K27cr in chromatin. Remarkably, the transcription factor associated with proto-oncogenes, MYC, recruits this complex to repress gene expression within chromatin subsequently. Furthermore, their experiments on mice revealed that either the knockdown of GAS41 or the deletion of H3K27cr binding can contribute to the inhibition of tumor growth. This sheds light on the novel role that histone cr plays in tumorigenesis.^215^

#### Hepatitis B virus-related hepatocellular carcinoma(HCC)

A study involving patients with hepatitis B virus-related HCC revealed an increase in histone H3 cit levels, which is closely associated with Beclin1 mRNA expression, vascular invasion, and serum AFP levels, reflecting a critical aspect of liver cancer progression.^216^

Significantly, HAT1, conventionally acknowledged as a HAT, has been additionally recognized for its succinyltransferase activity. In their quantitative proteomic analysis of HepG2 cancer cells, Yang and colleagues discovered that HAT1 coordinates the succinylation of a wide array of proteins, including histones and non-histone proteins. Furthermore, their investigations revealed that HAT1 is capable of H3K122succ, subsequently promoting favorable gene expression patterns within cancer cells. Clinically, elevated HAT1 levels have been observed in various cancerous tissues, such as HCC, pancreatic carcinoma, and cholangiocarcinoma. Therefore, it can be inferred that the succinyltransferase function of HAT1, coupled with the succ of PGAM1 mediated by HAT1, stands as a critical mechanism driving tumor progression.^217^

In 2016, Zhao et al. revealed that 2-hydroxybutyric acid can act as a foundation for the bhb of histones, activating gene expression. Intrinsically, β-hydroxybutyrate is a primary component of ketone bodies. This significant elevation in bhb levels effectively links gene expression to ketone body metabolism.^218^ Tumor cells demand substantial energy for growth, and in contrast to normal cells, this energy predominantly comes from glycolysis.^219^ Existing studies indicate that β-hydroxybutyrate or β-hydroxybutyrate-induced HPTMs may have pivotal roles in the onset and treatment of tumors. Scientists have observed that the build-up of β-hydroxybutyrate followed by the elevated presence of H3K9bhb in living organisms, mediated by MTA2, can initiate a cascade response fostering the progression of HCC. Additionally, it has been noted that the upregulation of the genes JMJD6, GREB3, GTPBP4, NPM1 and TIMM23 can affect the prognosis of HCC patients.^220^

#### Gastric cancer

In a distinct study, Zheng and colleagues provided persuasive findings showing that PADI2 plays a crucial role in promoting angiogenesis, cellular growth, movement, and influencing the tumor immune environment. This is achieved by amplifying the expression of CXCR2, KRT14 and TNF-α, thereby facilitating the onset of gastric cancer.^221^ Additionally, another study vividly showcased the close association between group mono-leuco-citrullination and IPO-38, emphasizing the potential of the latter as a biomarker for early gastric cancer detection.^222^

#### Prostate cancer(PCa)

In PCa research, Wang and colleagues discovered the crucial function of PADI2 in cell viability and cell cycle advancement. Their findings indicate that PADI2 facilitates the growth of prostate cancer cells.^223^ In a separate investigation, it was determined that PAD2 influences breast cancer by modulating histone cit.^224^

The research led by Xu and his team has accentuated the role of histone cr in PCa, uncovering that levels of histone cr are significantly elevated in PCa tissues compared to adjacent normal tissues and are closely associated with the severity of the disease. Through immunohistochemical analysis of 72 PCa patient samples and laboratory assays on three human PCa cell lines—including quantification by Western blot, as well as assessments of cell proliferation, migration, and invasion—the study unveiled potential mechanisms of histone cr in cancer progression. These findings suggest a significant correlation between the expression of histone cr and the clinical staging and grading of PCa, indicating its potential as both a prognostic marker and a therapeutic target.^225^

#### Pancreatic ductal adenocarcinoma

Histone succ is modulated by KAT2A, an enzyme exhibiting both lysine acetyltransferase and succinyltransferase functionalities. Investigations have revealed that KAT2A is not only abundantly manifested in human pancreatic ductal adenocarcinoma but also displays a positive correlation with the progressed stages of pancreatic ductal adenocarcinoma and a diminished patient survival rate. It is demonstrated that KAT2A augments the migration and invasiveness of pancreatic ductal adenocarcinoma cells through the regulation of 14-3-3ζ and β-catenin expression.^129^ The nuclear-localized α-ketoglutarate dehydrogenase complex in human cells has been identified to associate with KAT2A at the gene’s promoter region. KAT2A has the capability to execute succ on lysine 79 of histone H3. Acting as a succinyltransferase, KAT2A induces succ of histone H79 at lysine 3, predominantly occurring in proximity to the gene’s transcriptional initiation site. Experimental findings suggest that by obstructing the nuclear entry of the α-ketoglutarate dehydrogenase complex or suppressing the expression of KAT2A (Tyr645Ala), one can diminish gene expression, which consequently reduces the proliferation of tumor cells and overall tumor expansion.^126^ Concurrently, studies have shown that genomic instability is a hallmark of cancer.^226^ This association between histone succ and DNA damage has been underscored by several studies.^227^ To sum up, research has demonstrated that histone succ, regulated by KAT2A or HAT1, is pivotal in governing gene expression. This regulation significantly contributes to the growth, advancement, and metastasis of cancer cells.

Lu and his team, utilizing liquid chromatography and LC-MS/MS techniques, have for the first time identified histone hib sites in patients with pancreatic cancer. They discovered that histone modifications involving hib affect key metabolic pathways such as glycolysis, gluconeogenesis, and the TCA cycle, highlighting the significance of hib in the metabolism of pancreatic cancer. Moreover, the inhibition of Tip60 significantly suppresses the growth, migration, and invasion of pancreatic cancer, which correlates with the downregulation of hib. These results suggest that histone hib modifications are closely linked to the progression of pancreatic cancer.^228^ Additionally, predictive analyses using IPA software have revealed the role of actin cytoskeleton regulatory pathways in the development of oral squamous cell carcinoma, pointing to the regulation of actin assembly and stability as potential key mechanisms in cancer progression.^229^

#### Non-small cell lung cancer(NSCLC)

Furthermore, Tanikawa and his team observed in NSCLC that the levels of histone H4R3 cit are inversely proportional to p53 expression and tumor size, and that the p53 pathway, mediated by PADI4, significantly affects tumorigenesis.^230^

In essence, the citrullination-induced NET seems to ominously forecast a grim prognosis in the realm of cancer. An extensive analysis with long-term patient follow-up in cases of cancer-related VTE revealed a significant correlation: the cit status of histone H3 was strongly associated with VTE. This discovery strongly underscores the vital importance of NETs in the progression of VTE within oncogenic settings.^231^ Similarly, another investigative endeavor revealed an intriguing observation: elevated plasma concentrations of the NET biomarker, citrullinated histone H3 (H3cit), were concomitant with the onset of VTE in individuals diagnosed with pancreatic and lung cancers.^232^ In preliminary studies, researchers have observed a marked elevation in the levels of citrullinated H3cit in the plasma of patients with advanced cancer, and this elevation is tightly associated with the patients’ prognosis. The significant increase in H3cit levels nearly doubled the short-term mortality risk for patients, strongly highlighting its immense potential as a prognostic biomarker for cancer.^233^

#### Prolactinomas

For instance, DeVore and colleagues discovered that in prolactinomas, the activity of PAD2 and PAD4 and the levels of histone cit are elevated, promoting tumor growth. This process facilitates tumor development and proliferation through the downregulation of microRNAs, such as let-7c-2 and 29c.^234^

#### Others

In another investigation, core histone lysine bu sites, such as H3K18, H3K23, H3K79 and H4K77, were identified in esophageal squamous cell carcinoma cell lines.^12^

It has been shown that histone SUMOylation can function as a transcriptional repressor. This repression is primarily achieved either by disrupting histone ac or by interfering with the ubiquitination of H2BK123 through Rad6 and Bre1.^131^ The SUMO system comprises the activation enzyme E1, the conjugation enzyme E2 and the ligating enzyme E3. The E1 and E2 enzymes are limited in variety—E1 encompasses SAE1 and SAE2, while UBC9 is the sole E2 enzyme. In contrast, E3 enzymes possess a more extensive range owing to their diverse specificities, which facilitate binding to various substrates.^235^ Existing scholarly works indicate that Ubc9 holds a key role in regulating processes such as the cell cycle, cell growth, mitotic division, programmed cell death and DNA restoration. The direct correlation of Ubc9’s role in tumors histone SUMOylation remains somewhat nebulous. Nevertheless, the SUMOylation of multiple proteins is ascertained to significantly influence tumorigenesis. Significantly, increased levels of Ubc9 have been detected in a range of cancers such as lung,^236^ prostate,^237^ ovarian,^238^ bladder cancer^239^ and melanoma.^240^ One research demonstrates that inhibiting UBC9 curtails tumor development in mouse xenograft models, while the depletion of Ubc9 fosters STAT4-mediated macrophage activation. This activation, coupled with enhanced macrophage-CD8 + T-cell communication, serves to thwart tumor progression.^237^ The connection between SUMOylation and the onset of cancer is widely acknowledged, and continued investigation in this area is expected.

P53, recognized as a pivotal tumor suppressor, undergoes modification through bhb at lysines 120, 319 and 370. Under conditions of starvation, researchers noted an elevation in 2-hydroxybutyric acid serum concentrations in mice, accompanied by a rise in p53 bhb. This process hampers the ac of p53, leading to the cessation of cellular proliferation and a reduction in programmed cell death.^148^ This suggests to us the role of bhb in ketone metabolism and tumor management.

The role of histone modifications in cancer is multifaceted: different types of cancer, and even individual cases within the same cancer type, may exhibit distinct patterns of histone modifications. This underscores the highly heterogeneous nature of cancer as a disease, necessitating further research to better harness the potential of histone modifications in treatment and prognostic applications.

### Infectious diseases

Infectious diseases are complex biological processes closely associated with autoimmunity and inflammation. During the course of infection, microbes can induce epigenetic modifications, such as the suppression or promotion of expression of various inflammasomes.^241,242^ Numerous reports have mentioned the pivotal role of HPTMs such as phosphorylation, acetylation, or methylation in the pathogenesis of viral infections—including Herpes Simplex Virus, Hepatitis B Virus, and Human Immunodeficiency Virus (HIV)—as well as in sepsis models. However, research on the mechanisms related to novel HPTMs in the context of infectious bacterial or viral diseases remains scant.^243–245^ Research has found that all subjects, including healthy volunteers, express H3K18la; however, individuals with septic shock exhibit the highest levels. This suggests that H3K18la may reflect the severity of critical illness and the presence of infection. H3K18la could potentially regulate the anti-inflammatory function of macrophages in sepsis by promoting the expression of inflammatory cytokines and the overexpression of Arg1.^246^

Studies have demonstrated that by augmenting the expression of ACSS2 (Acyl-CoA Synthetase Short-chain Family Member 2), histone cr can be induced, thereby reactivating latent HIV. Pharmacological inhibition or siRNA-mediated knockdown of ACSS2 can reduce HIV replication and activation. Moreover, when used in conjunction with protein kinase C agonists or histone deacetylase inhibitors, ACSS2 can efficiently reactivate latent HIV. In simian models of HIV, an increase in ACSS2 expression correlates with alterations in fatty acid metabolism. This research links ACSS2 with HIV latency and offers a potential new target for HIV eradication strategies.^116^ The study by Jiang et al. suggests that histone cr is an epigenetic modification present on the long terminal repeats of HIV, which modulates the transcription and latency of the virus. Inhibition of cr can prevent the reactivation of latent HIV, indicating that the suppression of cr could contribute to the maintenance of HIV latency, offering a potential target for controlling and reducing the incidence of AIDS.^116^

### Cardiovascular diseases

Recent research findings indicate that histone la is critical for the preservation of cardiac sarcomere structure and function. This modification is specifically directed at the α-myosin heavy chain (α-MHC), which is a major contractile protein in the myocardium. La strengthens the interaction between α-MHC and titin^247^—a key sarcomeric protein that supports the elasticity and integrity of muscle tissue—thereby la contributes to maintaining myocardial contractile function. Scholars hypothesize that augmenting α-MHC la could emerge as an innovative therapeutic approach for combating heart failure. This hypothesis suggests that focusing on α-MHC la presents a potential new avenue for treatment strategies in heart failure management. Myocardial infarction triggers a complex inflammatory response, which is crucial for the control of acute damage and subsequent cardiac repair.^248^ The initial cellular response encompasses a systemic emergency hematopoiesis reaction coupled with the swift mobilization of neutrophils and monocytes to the site of action.^249,250^ A sustained excessive inflammatory response can exacerbate myocardial damage and cardiac dysfunction. The prompt initiation of reparative signaling within monocytes and macrophages is critical for the expeditious re-establishment of immune equilibrium and the commencement of the healing process post-myocardial infarction.^251,252^ Research suggests that histone la can influence the anti-inflammatory and pro-angiogenic behavior of monocyte-macrophage cells, thereby promoting the transcription of genes associated with repair. This contributes to a healing milieu and enhances cardiac performance post-myocardial infarction. These insights uncover the pivotal function of IL-1β-induced recruitment of GCN5 (General Control Non-repressed 5) to histone H3K18la, shedding light on its possible role as a regulatory precursor for monocyte histone la and the subsequent expression of genes involved in repair following myocardial infarction.^253^

**Fig. 7:**
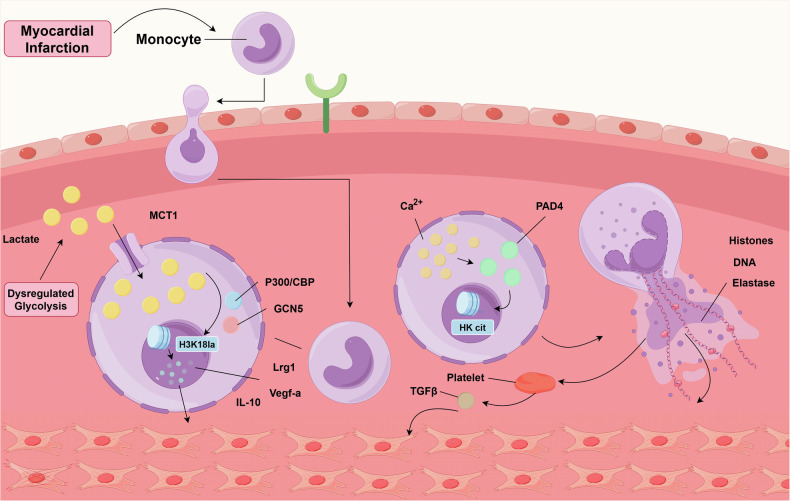
Novel HPTMs: dual roles in cardiac repair and fibrosis. When MI occurs, monocytes undergo metabolic reprogramming, leading to dysregulated glycolysis and consequently increased lactate production. The generated lactate is transported into monocytes via MCT1 and accumulates intracellularly, providing the substrate for histone la. Upon histone la, specific reparative genes such as Lrg1, Vegf-a, and IL-10 are activated and expressed. The expression of these genes modulates the dual anti-inflammatory and pro-angiogenic activities of monocytes, ultimately promoting cardiac repair. In additional, the activation of PAD4 is triggered by an increase in calcium ion concentration, leading to histone cit and the formation of NETs. NETs are composed of DNA, histones, and antimicrobial proteins and are released into the extracellular space. These NETs promote inflammatory responses, damage cardiomyocytes and endothelial cells, and interact with platelets, promoting the release of TGFβ, which in turn promotes myofibroblast fibrosis. NET formation and fibrosis are increased, whereas mice lacking PAD4 exhibit reduced fibrosis and maintain good cardiac function

Vascular diseases are associated with changes in PAD activity and citrullinated proteins.^254–261^ In cardiomyocytes and fibroblasts, PAD2 and PAD4 have been identified as the predominant isoforms.^262^ HPTMs of myofibrillar proteins in cardiomyocytes, such as cit, phosphorylation, oxidation, and ac, can lead to alterations in the structure and function of these proteins, resulting in diminished cardiac contractility.^262^ In ischemic heart diseases, there is a marked increase in the cit of myosin heavy chains, and in patients with heart failure, there is an elevated cit of cardiac contractile proteins by PAD2.^262^ PAD4 induces the formation of NETs, activates platelets, and promotes the secretion of transforming growth factor-beta (TGF-β), ultimately contributing to cardiac fibrosis.^263,264^ Moreover, the absence of PAD4 has been shown to confer cardioprotection in mice.^263^ Extracellular DNA or NETs adversely affect cardiac function following acute myocardial infarction injury.^265^ Chromatin remodeling is crucial for controlling gene expression and cardiac growth in response to acute and chronic stimuli.^266,267^ Moreover, in various models of deep vein thrombosis, it has been demonstrated that NETs can instigate coagulation. NETosis, a form of inflammatory programmed cell death, has the potential to precipitate harmful pathologies, including small vessel vasculitis, sepsis, systemic lupus erythematosus, and deep vein thrombosis, illustrating its significant yet potentially detrimental role in these conditions^257,268,269^ **(**Fig. 7**)**.

### Metabolic disease

An increasing body of research suggests that epigenetic mechanisms mediated by histone modifications may constitute a potential etiology for Type 2 Diabetes. In a prediabetic state, researchers conducted a systematic analysis of HPTMs of histones in the liver of a mouse model of obesity induced by a high-fat diet. They uncovered novel alterations in modifications such as ac, bu, ma, and succ, hinting that these recently revealed histone ac events may play a latent role in the development and progression of diabetes and obesity. Notably, the study also found that metformin could reverse certain specific histone modification markers, such as changes in histone H3K36ma2.^15^ Research indicates that levels of malonyl-CoA are elevated in the muscles of obese and type 2 diabetes patients, underscoring the possibility that certain histone modifications may be intricately linked with the progression of these conditions.^270^ In particular, bhb has been identified as a marker of active genes during starvation or diabetes ketosis induced by streptozotocin, with this modification closely associated with the metabolic pathways of the starvation response. Additionally, ketogenesis stimulated by starvation increased both bhb and bu, while the observed rise in malonylated proteins in a diabetes mouse model affected glucose and fatty acid metabolic pathways, suggesting that these modifications could potentially reverse insulin resistance.^17,271,272^

The ablation of Pkm2 in endothelial cells leads to diminished serum lactate levels, which subsequently influences histone la within bone marrow stromal stem cells (BMSCs). This particular la modification is crucial for the differentiation of BMSCs into osteoblasts, which are the principal cells responsible for bone synthesis. Histone H3K18 la modulates several genes vital for osteogenesis, such as type I collagen α2 chain (COL1A2), cartilage oligomeric matrix protein, ectonucleotide pyrophosphatase/phosphodiesterase 1 (ENPP1), and transcription factor 7-like 2. Therapeutic strategies such as the upregulation of PKM2 in endothelial cells, exogenous lactate supplementation, and physical exercise have shown efficacy in reversing the compromised phenotype in mice with a deficit in endothelial PKM2, indicating promising avenues for osteoporosis treatment.^273^

### Reproductive disease

Emergent HPTMs are associated with sperm maturation impairments and developmental anomalies that may impact male fertility. The bromodomains of BRDT can recognize histone ac and recruit transcription complexes to chromatin, thereby promoting the expression of specific genes.^274^ Studies suggest that ac and bu at H4K5 and H4K8 positions compete at gene promoters bound by highly active BRDT, with bu also marking the delayed removal of histones during late spermatogenesis.^72^ Research has unveiled that the chromodomain Y-like transcriptional corepressor (CDYL) acts as a negative regulator of histone cr. Functioning as a crotonyl-CoA hydratase, CDYL catalyzes the conversion of crotonyl-CoA to β-hydroxybutyryl-CoA, effectively diminishing the pool of crotonyl-CoA available for the crotonylation of histone lysine residues. The function of CDYL-mediated histone cr regulation is particularly significant in spermatogenesis. Research indicates that dysregulation of histone cr in CDYL transgenic mice leads to decreased male fertility, characterized by reduced epididymal sperm counts and impaired sperm motility. The study suggests that histone cr regulation by CDYL might be associated with spermatogenic failure in infertile males with AZFc deletions. This link may provide a new avenue for exploring therapeutic strategies or diagnosing certain types of male infertility.^114^ There exists a clear necessity for additional studies to deepen our comprehension of how novel HPTMs might influence reproductive anomalies.

### Neuropsychiatric disease

Epigenetic modifications have been found to be of significant importance in neurological conditions such as PD, Huntington’s disease, AD, and Major Depressive Disorder (MDD).^275–277^ Contemporary studies propose that the onset of MDD may be intricately tied to several physiological and biochemical phenomena. These include the activation of the corticotropin-releasing hormone (CRH)/hypothalamic-pituitary-adrenal axis, excessive stimulation of the sympathetic nervous system, irregular secretion patterns of monoaminergic neurotransmitters, increased production of pro-inflammatory cytokines, diminished levels of neurotrophic factors, and significant epigenetic modifications.^278–283^ Epigenetics may serve as one of the bridges linking environmental and genetic factors. Stressful events can lead to alterations in epigenetic modifications, thereby causing changes in gene expression.

MDD emerges as a complex condition influenced by a broad array of factors, including environmental, genetic, psychological, and biological determinants. Those afflicted with MDD may endure profound symptoms that encompass enduring and intense feelings of sadness and desolation, cognitive impairments, anhedonia, diminished verbal and motor activity, along with disruptions in sleep patterns.^284,285^ Contemporary research indicates that significant stress events can precipitate modifications in histone configurations within the human brain, catalyzing transcriptional alterations that may culminate in the onset of MDD. Histone ma, ac, phosphorylation, cr, and bhb have all been identified as modifications intricately linked with the pathogenesis of MDD. Of particular interest in our discussion are the relationships between histone cr, bhb, and MDD.^286^ The investigation conducted by Liu and colleagues illuminates that MDD, precipitated by sustained social defeat stress, correlates with a diminished presence of histone crotonylation. This reduction is observed alongside the upregulated expression of CDYL, suggesting a potential mechanistic interplay in the manifestation of MDD. This study is the first and, to date, the only one that demonstrates the link between MDD and histone crotonylation. Initially, researchers discovered that β-hydroxybutyrate, as a ketone body, plays a significant role in neuro-related disorders. It was found that bhb can protect neurons from toxic damage and prevent the degenerative changes in dopaminergic neurons seen in Alzheimer’s and Parkinson’s diseases.^146,147^ Recent research suggests that β-hydroxybutyrate may possess antidepressant effects for Major MDD induced by chronic unpredictable stress.^287–289^ Chen and colleagues were the first to associate the antidepressant effects of β-hydroxybutyrate with histone modifications. In mice with MDD induced by spatial restriction stress, they observed a reduction in the levels of H3K9bhb. Injections of β-hydroxybutyrate were found to increase both β-hydroxybutyrate and H3K9bhb levels, as well as enhance the expression of brain-derived neurotrophic factor (BDNF).^290^ This deduction posits that H3K9bhb could serve as a pivotal regulatory factor for the expression of BDNF, hinting at the role of histone bhb as a significant avenue for unraveling the underpinning mechanisms of MDD.

At present, the pharmacological treatment of depression in clinical settings classifies antidepressants into seven primary categories: selective serotonin reuptake inhibitors (SSRIs), serotonin antagonists and reuptake inhibitors, serotonin-norepinephrine reuptake inhibitors, norepinephrine-dopamine reuptake inhibitors, tricyclic antidepressants, monoamine oxidase inhibitors, and melatonergic antidepressants. Each class operates via distinct mechanisms to modulate neurotransmitter systems and alleviate depressive symptoms.^291^ These medications chiefly exert their effects by inhibiting the activity of serotonin and norepinephrine. However, approximately 40% of MDD patients exhibit insensitivity to these drugs. Consequently, the development of more effective and less toxic antidepressant medications is imperative.^292^ Furthermore, the emerging focus among researchers on antidepressants that influence histone modifications, especially HDAC inhibitors, highlights a novel approach in the therapeutic landscape of MDD. These inhibitors have shown promise in alleviating symptoms of MDD by modulating epigenetic mechanisms. Bhb may be a critical factor in ketogenic diets, exhibiting broader and more pronounced neuroprotective effects in ameliorating refractory epilepsy compared to traditional restrictive diets.^293^

### Others

In the study of pulmonary fibrosis, it was found that lung myofibroblasts, under the stimulation of TGF-β1, exhibit increased glycolytic activity, leading to the substantial production of lactate. This lactate accumulates not only in vitro cultures but is also detected in mice with a model of pulmonary fibrosis. Lactate promotes histone la via p300, enhancing the expression of pro-fibrotic genes and propelling the progression of lung fibrosis. Additionally, exposure to PM2.5 augments glycolytic activity, increasing the generation of lactate and other metabolic byproducts. The activation of the TGF-β/Smad2/3 and VEGFA/ERK pathways instigates the development of pulmonary fibrosis. The application of an LDHA inhibitor, specifically GNE-140, as a pre-treatment strategy, has been shown to significantly mitigate pulmonary inflammation and fibrosis triggered by PM2.5, showcasing its therapeutic promise in addressing such conditions.^294,295^

Renal disorders can be categorized into acute kidney injury (AKI) and chronic kidney disease (CKD), with a subset of CKD potentially progressing to end-stage renal disease. Histone cr has been observed in renal tubular cells of healthy mouse and human kidney tissues. During acute AKI, levels of histone cr in renal tissues are elevated. This finding is replicated in vitro in cultured renal tubular cells exposed to the cytokine TWEAK, suggesting that TWEAK may be one of the factors influencing histone cr levels. Experiments involving the administration of crotonate to cultured renal tubular cells or kidneys found that crotonate treatment increased the expression of PGC-1α and sirtuin-3, and decreased the expression of CCL2. Systemic administration of crotonate in animal models can prevent the onset of AKI, maintain renal function, and prevent the decrease in PGC-1α and sirtuin-3 levels, as well as the increase in CCL2 expression.^14^ Furthermore, analysis based on the Kyoto Encyclopedia of Genes and Genomes (KEGG) suggests that proteins modified with hib are enriched in the IL-17 signaling pathway and in categories related to phagosomes. These pathways and categories are considered to be significantly associated with IgAN. The data imply that hib modifications may play a crucial regulatory role in the development and progression of IgAN.^296^ Chen et al. measured the global levels of cr sites within the proteome of patients with CKD and those undergoing maintenance hemodialysis, finding decreased levels of cr in dialysis patients and increased levels in CKD patients. This suggests that cr may play a significant regulatory role in the transition from AKI to CKD.^297^ The research conducted by Shimazu and colleagues presents groundbreaking findings on the linkage between β-hydroxybutyrate and renal ailments. It was revealed that β-hydroxybutyrate significantly shields murine kidneys from oxidative stress. This protective effect is achieved through the inhibition of HDAC activity and the augmentation of histone ac at the promoters of Foxo3a and Mt2, thereby unveiling a potential therapeutic avenue for renal diseases.^297^

Research indicates that Salvianolic acid B can downregulate the expression of lactate dehydrogenase A (LDHA), thereby inhibiting histone la in macrophages, which effectively mitigates carbon tetrachloride-induced liver injury. By examining liver tissues and isolated Kupffer cells, it has been confirmed that Sal B affects the M1 polarization and histone la levels in macrophages.^298^

**Fig. 8:**
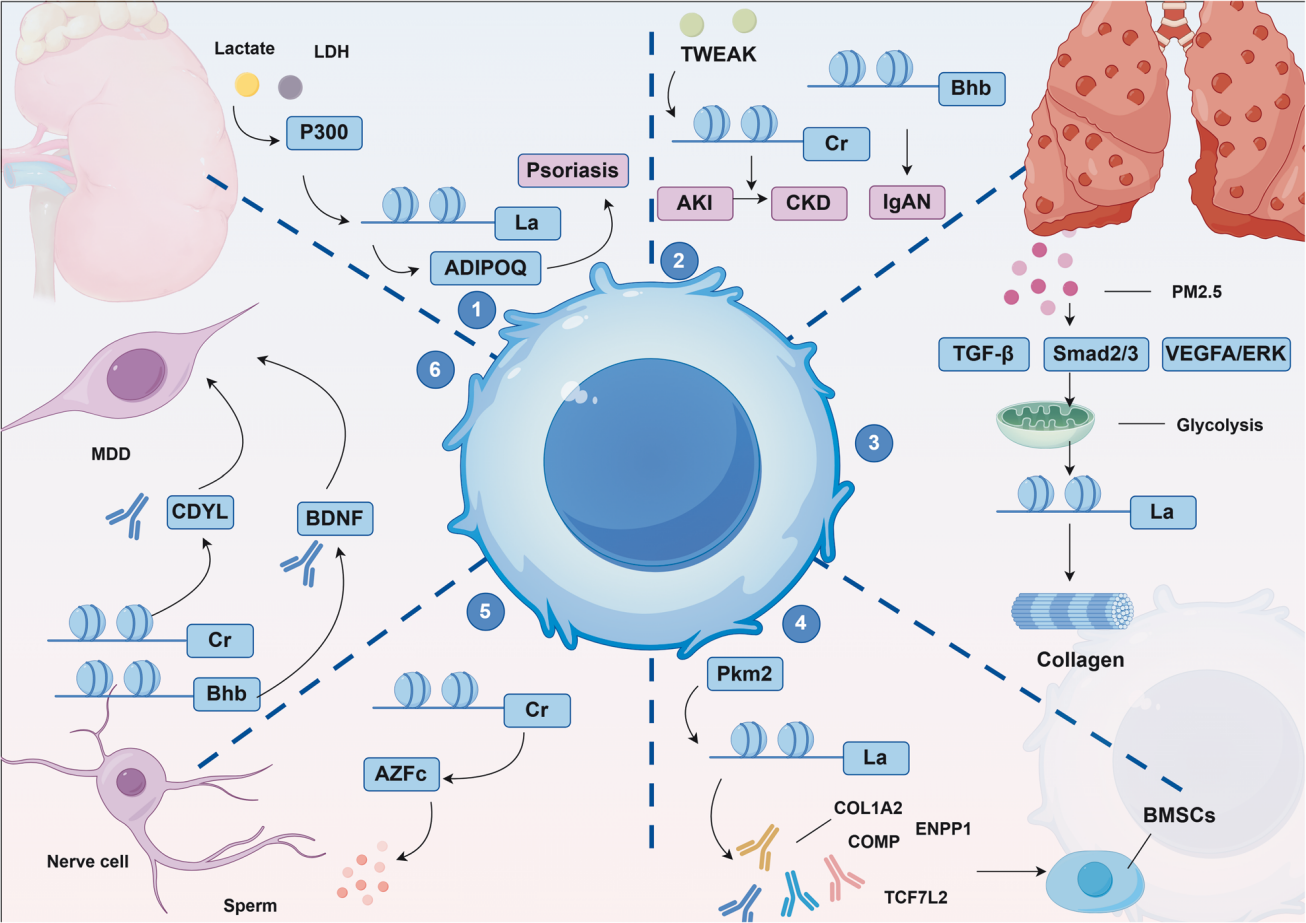
Impact of novel HPTMs on various disease pathogenesis. The figure represents the roles of novel HPTMs in different diseases. In MDD, for example, the expression of CDYL was increased and pr was inhibited; H3K9bhb and BDNF had an antidepressant effect. Spermatogenic failure is caused by the dysregulation of histone cr in AZFc deletions of infertile males. Under expression of Pkm2 regulates COL1A2, COMP, ENPP1, and TCF7L2, through histone la involving BMSCs osteoblast differentiation. In response to IL-17 and TNF stimulation, LDHA- and LDHB-mediated lactate production positively regulates histone la acetylation by inducing p300-dependent ADIPOQ protein and promoting the gene expression of pro-fibrotic genes, exacerbating the pathogenesis of psoriasis. In lungs, PM2.5 activates the TGF-β/Smad2/3 and VEGFA/ERK pathways, upregulating lactate-generating glycolysis in lung myofibroblasts through histone la to encode for collagen. The TWEAK cytokine induced cr expression, and cr had a significant effect on the regulation of the AKI-CKD transformation. The change of hib was a key regulatory action for IgAN

In the progression of psoriasis, numerous studies have highlighted that elevated levels of lactate or lactate dehydrogenase are key factors.^299,300^ Research has shown that elevating global la and H3K18la levels can increase the levels of the adiponectin ADIPOQ protein,^301–303^ whereas transfection with si-LDHA reduces ADIPOQ protein levels. In the skin tissues of psoriasis patients, ADIPOQ levels are significantly reduced, and ADIPOQ possesses the potential for diagnosing psoriasis. Furthermore, the study uncovered that the downregulation of H3K18la levels inhibits the transcriptional activity of the ADIPOQ gene, which is a key factor contributing to the diminished ADIPOQ levels in psoriasis patients ^304^(Fig. 8).

## Targeted therapy of Novel Hptms

In this section, we will categorize the current inhibitors involved in HPTMs into three groups, summarizing their mechanisms of action and clinical applications. Additionally, we will specifically collate inhibitors that are still in the experimental stage (Table 2).

**Table 2:** Novel histone modifications: mechanisms, signaling pathways, and therapeutics in various human diseases

Diseases type	Histone modifications	Inhibitors targeting histone modifications	Reference
Bladder cancer	Lactylation	1. YTHDF2 enhances BLCA cell growth in vitro and in vivo and may improve BCG therapy response.^489^ 2. The circXRN2-Hippo pathway helps control bladder cancer progression by inhibiting H3K18 acetylation and managing LCN2 expression.	^489^
Lung cancer	SUMOylation	Ubc9, crucial for SUMOylation, is found at higher levels in lung cancer. Blocking Ubc9 slows tumor growth in mouse models, and reducing Ubc9 boosts macrophage activity, leading to enhanced macrophage and CD8 + T cell interaction, which helps stop tumor growth.	^397^
	Crotonylation	HDAC1-induced caspase-1 cr affects NSCLC PEM sensitivity through GSDMD.^397^	
	Citrullination	Numerous studies indicate that in lung cancer, the expression of PAD-mediated histone cit significantly elevated.	
ccRCC	Lactylation	1. In vivo studies show correcting abnormal histone la greatly reduces ccRCC growth and spread. Targeting histone la and PDGFRβ together can significantly improve treatment outcomes.^209^ 2. Disrupting the reinforcing loop between histone lysine acetylation and PDGFRβ signaling may present an innovative approach for treating patients with ccRCC. Simultaneously targeting histone lysine acetylation and PDGFRβ has the potential to significantly enhance therapeutic outcomes.^209^	^209^
Breast cancer	Lactylation	1. Inhibiting glycolytic enzymes affects c-Myc-SRSF10 axis, lowering breast cancer cell proliferation. Kla is key in breast cancer drug resistance and prognosis.^189^ 2. Intestinal bacteria-derived lipopolysaccharides increase LINC00152 expression through histone la induction, boosting breast cancer cell migration and invasion by weakening suppressive protein YY1’s binding.	^189,351^
	Citrullination	The PBA-PAD4 inhibitor effectively hinders the proliferation and dissemination of breast cancer cells and significantly reduces the development of NETs within cancerous tissues.^351^	
Prostate cancer	Lactylation	Lactate inhibition activates TAM anti-cancer effects via histone la in prostate cancer. Numb/Parkin pathways and evodiamine targeting histone la offer therapeutic promise. Combined therapy with PI3K and MEK inhibitors reduces tumor growth by targeting H3K18la in TAMs, effectively managing AVPC.^187,352,490^	^187,225,352,490^
	Crotonylation	1. PCa linked to histone cr; Compounds like I-BET762, I-BET726, and CPI-203 target BRD4 to curb prostate cancer cell proliferation, movement, and invasion.^225^ 2. Using the PADI inhibitor Cl-Amidine with the AR inhibitor enzalutamide significantly reduces CRPC cell growth in vitro and tumor growth in vivo. Studies underline PADI2’s crucial role in prostate cancer, especially CRPC, indicating its potential as a therapeutic target.	
	SUMOylation	Ubc9, crucial for SUMOylation, is found at higher levels in prostate cancer. Blocking Ubc9 slows tumor growth in mouse models, and reducing Ubc9 boosts macrophage activity, leading to enhanced macrophage and CD8 + T cell interaction, which helps stop tumor growth.	
HCC	Lactylation	1. CCNE2 la in HCC promotes growth, while SIRT3 activation by honokiol induces cell death via CCNE2 Kla modification. NR6A1, OSBP2, UNC119B genes may be new targets in HCC treatment resistance. RJA targets glycolytic lactate to modify H3 la sites, providing anti-tumor effects. The CENPA-YY1-CCND1/NRP2 axis offers new HCC therapy approaches. DML blocks HCC by targeting LCSC-induced tumorigenicity at H3K9la and H3K56la sites.^410,411,491–493^ 2. The triterpenoid compound DML targets two tumor-promoting sites, H3K9la and H3K56la, effectively slowing HCC growth. Its mechanism was confirmed in a nude mouse tumor model, suggesting DML as a promising HCC treatment. 3. RJA impedes HCC progression by disrupting lactate production and inhibiting la at histone sites H3K9la and H3K14la.	^410,411,491–493^
	citrullination	Numerous studies indicate that in HCC, the expression of PAD-mediated histone cit is significantly elevated.	
	Succinylation	1. Aspirin slows down liver cancer growth by decreasing PGAM1 succ, which disrupts cancer cells’ energy production. Aspirin is found to suppress sugar breakdown in cancer cells and boost its cancer-fighting effects by lowering ENO1 levels. 2. Research shows that blocking enzymes such as KAT2A that add succinyl groups to proteins can reduce tumor growth.	
	2-hydroxybutyrylation	β-hydroxybutyrate accumulation, driven by MTA2, raises H3K9bhb levels, accelerating HCC growth. Also, higher levels of genes like JMJD6 and GREB3 could influence HCC patient prognosis.	
Cholangiocarcinoma	Succinylation	Increased HAT1 levels in cholangiocarcinoma link to its role in promoting tumor growth, notably through its influence on PGAM1’s function.	
Oral squamous cell carcinoma	2-hydroxyisobutyrylation	Significant bib modifications in actin cytoskeleton proteins suggest that actin regulation might be a key mechanism in oral squamous cell carcinoma’s progression, as analyzed using IPA software.	
Gastric cancer	Citrullination	1. Histone cit, crucial for NETs formation, may be targeted to curb gastric cancer progression.^494^ 2. Numerous studies indicate that in gastric cancer, the expression of PAD-mediated histone cit is significantly elevated. 3. PADI2 is key in driving blood vessel formation, cell growth, and movement, and it shapes the immune environment in gastric cancer by increasing CXCR2, KRT14, and TNF-α levels, promoting cancer development. 4. The link between leukocyte cit and IPO-38 highlights IPO-38’s potential as an early gastric cancer biomarker.	^494^
Pancreatic cancer	Citrullination	1. A study found that in pancreatic patients, elevated plasma levels of the NET biomarker H3cit are linked with venous thromboembolism. Elevated H3cit levels in advanced cancer patients significantly correlate with worse prognosis and nearly double the risk of short-term mortality, highlighting its potential as a crucial cancer prognostic biomarker. 2. Evodiamine, a bioactive alkaloid, can effectively suppress the expression of histones and HIF1A in PCa cells, thereby inhibiting the lactate-induced angiogenesis process. Concurrently, it enhances the transcription of Sema3A and inhibits the transcription of PD-L1, collectively impeding the formation of vasculature and the growth of the tumor in prostate cancer.^352^ 3. The PAD inhibitor Cl-Amidine, in combination with the AR signaling inhibitor enzalutamide, produces a synergistic effect that significantly inhibits the proliferation of CRPC cells in vitro.^223^ 4. I-BET762, I-BET726, and CPI-203, by modulating the levels of BRD4, influence the dynamics of histone cr, thereby inhibiting the growth, mobility, and invasive behavior of PCa cell lines.^225^	^223,225,228,352^
	Succinylation	1. HAT1, which regulates protein succ, shows increased levels in pancreatic cancer tissues. 2. KAT2A, which has dual enzyme functions, is overexpressed in pancreatic cancer, linked to worse outcomes. It boosts cancer cell spread by controlling 14-3-3ζ and β-catenin levels.	
	2-hydroxyisobutyrylation	Studies show Tip60 inhibitors effectively slow pancreatic cancer’s growth, migration, and invasion by lowering cellular bib levels, highlighting bib’s key role in the disease’s aggressiveness.^228^	
CRC	Lactylation	NDGA is capable of directly binding to RARγ and inhibiting the TRAF6-IL-6-STAT3 signaling pathway, offering a novel therapeutic strategy targeting tumor-promoting macrophages in CRC.^406^	^406,408,409^
	Butyrylation	The antitumor efficacy and high specificity of largazole-7 may be attributed to its targeted action on histone deacetylases in CRC cells. Moreover, studies indicate that when the cancer therapeutic ligand GnRH-III is conjugated with deacetylase inhibitors, it enhances the tumor suppressive effects and exhibits greater binding affinity.^408,409^	
Glioblastoma	Crotonylation	1. GCDH increases histone lysine cr via CBP. Lower cr raises cell RNA/DNA, activating MDA5/cGAS and interferon signaling, inhibiting GSC tumorigenicity and boosting CD8 T cell activity.^412^ 2. Glioblastoma stem cells enhance crotonyl-coenzyme A and H4 lysine cr by altering lysine breakdown.	^412^
Multiple myeloma	Citrullination	BMS-P5* delays multiple myeloma onset and progression in mice, suggesting PAD4 as a therapeutic target.^353^	^353^
Lymphoma	Butyrylation	SAHA, a drug for cutaneous T-cell lymphoma, is also effective against neuroblastoma, increasing histone bu and ac.	
Leukemia	Propionylation	Specific antibodies revealed pr in leukemia cells U937, showing a notable decrease in H3K23 pr. This suggests histone pr’s role in leukemia’s development, potentially marking it as a diagnostic indicator.	
Infectious diseases	Citrullination	1. The PAD inhibitor YW3-56 can alleviate inflammation and damage caused by LPS, offering a novel therapeutic strategy for treating endotoxic shock and related inflammatory conditions.^355^ 2. The second-generation PAD inhibitor, BB-Cl-amidine, has the potential to modulate T-cell immune responses, thereby mitigating the severity of arthritis inflammation.^337^	^337,355^
	Lactylation	1. The concurrent use of HDAC inhibitors with common antidepressants, such as fluoxetine, can significantly reduce behaviors associated with MDD.^388^ 2. HDAC inhibitors can also alleviate the neurotoxicity of α-synuclein, thereby ameliorating the symptoms of Parkinson’s disease.^381^ 3. The clinically approved antiepileptic drug Stiripentol, capable of crossing the blood-brain barrier, inhibits the activity of LDHA/B. As a lactate inhibitor, it renders GBM cells more susceptible to Resistance to TMZ both in vitro and in vivo.^179^	^179,381,388^
Multiple sclerosis	Citrullination	Non-covalent inhibitors, based on α-amino acid and isocyanate ester core structures, effectively reverse the physical disability induced by experimental autoimmune encephalomyelitis and reduce T-cell infiltration in the brain.^354^	^354^
Liver fibrosis	lactylation	The expression of HK2 is regulated by lactylation-mediated histone modification of gene expression. Targeting HK2 can inhibit the activation of hematopoietic stem cells and ameliorate systemic liver fibrosis.^405^	^405^

*ccRCC* clear cell renal cell carcinoma, *CRC* colorectal cancer, *HCC* hepatocellular carcinoma, *NSCLC* non-small cell lung cancer, *CRPC* Castration-resistant prostate cancer, *YTHDF2* YTHDF2, an m6A reader protein, recognizes m6A marks on PER1 and TP53 mRNA when activated by histone lactylation, leading to their degradation, *UBC9* The SUMO system includes activating enzyme E1, conjugating enzyme E2, and ligating enzyme E3. UBC9 is the sole E2 enzyme, *HDAC1* histone deacetylase 1, *PAD* peptidyl-arginine deaminase (PAD) enzymes are involved in the cit of histones, *HAT1* histone acetyltransferase 1 (HAT1) is known for acetyltransferase activity but also has succinyltransferase activity, *KAT2A* KAT2A is an enzyme with both lysine acetyltransferase and succinyltransferase functions, *BMS-P5* BMS-P5 is a novel-specific small molecule inhibitor targeting peptidylarginine deiminase 4 (PAD4)

### Inhibitors targeting enzymes of novel HPTMs

#### SIRT inhibitors

Nicotinamide and its derivatives, such as nicotinamide riboside and nicotinamide mononucleotide, are important precursors of NAD + .^305^ Nicotinamide acts as an endogenous inhibitor of SIRTs and can inhibit SIRT1 and SIRT2.^306,307^ AK-7 is a selective SIRT2 inhibitor that has demonstrated improvements in behavioral and neuropathological phenotypes, extended lifespan, and ameliorated the neuropathology associated with HD in animal models.^308,309^ β-naphthol inhibitors encompass a variety of SIRT inhibitors that possess a β-naphthol structure, including splitomicin, sirtinol, salermide, HR-73, and cambinol.^310,311^ These compounds were discovered through in vitro cellular screenings; for instance, sirtinol and splitomicin are capable of inducing apoptosis and autophagy in cancer cells. A series of Sir2 inhibitors based on the indole structure were identified through large-scale fluorescent screening. These compounds, including EX-527, AC-93253, inauhzin, and Ro31-8220, predominantly inhibit SIRT1 and are associated with enhanced cell survival and p53 acetylation.^312,313^ SIRT The SIRT rearranging ligand SirReal2 is a potent SIRT2 selective inhibitor that acts by inducing structural rearrangement and interacting with an unknown binding site, leading to increased acetylation of chromatin protein H3.^314^ Discovered through phenotypic screening, tenovin-1 and its more water-soluble analog tenovin-6 primarily inhibit SIRT1 and SIRT2, decreasing tumor growth both in vitro and in vivo.^315^ Additional SIRT inhibitors, including a range of compounds such as suramin, aristoforin, AGK2, and Tripos 360702, have demonstrated the ability to inhibit SIRT activity. These compounds show potential in inhibiting cancer cell growth and modulating cell signaling pathways.^316–319^

While there have been significant advancements in the research of SIRT modulators over the past few decades, the studies have been uneven, and their clinical potential remains underexploited. Current investigations have adequately covered inhibitors of SIRT1 and SIRT2, yet research on inhibitors for SIRT3-7 is still lacking. Moreover, research on SIRT activators has primarily focused on SIRT1; there is a need for further exploration into activators and inhibitors for other SIRT family members to fully harness the therapeutic potential of SIRT molecules.^320^

#### PAD inhibitors

PADs are a group of active enzymes that catalyze the irreversible HPTMs of arginine to citrulline.^321,322^ Through this process, the structure and function of numerous target proteins are altered, including fibrinogen, TGF-β, nicotinamide N-methyltransferase, cytokines, and chemokines.^323,324^ Cit serves as a biomarker for various diseases, particularly in instances of dysregulated PAD activity.^321,322^ Five PAD isoforms, PAD1 through PAD4 and PAD6, have been identified in mammals.^325^ PAD1, PAD2, and PAD4 localize to both the cytoplasm and the nucleus, where they can citrullinate histones and other chromatin-associated proteins.^326^ All PAD isoforms, with the exception of PAD6, exhibit catalytic activity.^327,328^ The PAD family plays a role in regulating multiple biological processes, including cell differentiation, apoptosis, innate immune responses, embryonic development, myelination, and gene regulation.^329^

Inhibitors targeting PAD4 are delineated into two classes: irreversible inhibitors and reversible inhibitors. The irreversible category encompasses compounds such as F-amidine, o-Cl-amidine, BB-Cl-amidine, Cl-amidine, YW-356, o-F-amidine, Thr-Asp-F-amidine, and Thr-Asp-Cl-amidine. On the other hand, reversible inhibitors comprise agents like GSK199, streptonigrin, GSK484, along with select antirheumatic medications. These distinct groups of PAD4 inhibitors offer various therapeutic approaches for conditions involving this enzyme.^259^ The mechanism by which 2-fluoroacetamidine, Cl-amidine, and F-amidine exert their inhibitory effects involves a primary assault on the carbonyl carbon of the thiolate anion at Cys645. This action precipitates the creation of a protonated tetrahedral intermediate, noted for its stability, which underscores the inhibitory process of these compounds.^330^ The potency of o-F-amidine is 65 times that of F-amidine, with selectivity for PAD1 being at least sixfold higher than for PADs 2-4.^331^ This indicates that o-F-amidine has significant advantages in selectivity and potency. Despite the identification of numerous PAD inhibitors, the efficacy of most is limited.^332^ Variations exist among covalent PAD inhibitors in terms of in vivo stability, bioavailability, and isozyme selectivity, such as D-Cl-amidine (a selective inhibitor for PAD1), Cl4-amidine (selective for PAD3), and Thr-Asp-F-amidine (TDFA, a selective inhibitor for PAD4).^321^ Most reversible inhibitors, like minocycline, paclitaxel, and streptonigrin, are weaker PAD inhibitors, yet GSK484 and GSK199 are potent and selective inhibitors targeting PAD4.^333^ Cl-amidine, a haloacetamidine class PAD inhibitor, exhibits a higher selectivity for PAD4. It can prevent vascular abnormalities, arterial thrombosis, endothelial dysfunction, and aberrant vascular repair in systemic lupus erythematosus, as well as inhibit the formation of NETs, reduce the area of atherosclerotic lesions, and prolong carotid thrombus formation time in Apolipoprotein E knockout mice.^334^ Recent studies have demonstrated that PAD4 inactivation can protect murine hearts from damage after myocardial infarction/reperfusion injury.^265^ Furthermore, the Cl-amidine analog YW3-56, with improved bioavailability, can modify the gene encoding the upstream inhibitor of the mammalian target of rapamycin complex 1, SESN2.^335^ Treatment with Cl-amidine reduces histone citrullination in neutrophils and prevents NET formation.^336^ BB-Cl-amidine, a second-generation PAD inhibitor, alters T-cell immune responses and decreases the severity of inflammation in arthritis.^337^ Both Cl-amidine and BB-Cl-amidine are potent PAD inhibitors in various anti-tumor models.^336,338^

The development and application of PAD inhibitors represent a significant advancement in the field of cancer therapy, targeting the cit process, which is crucial in various cancer-related processes including tumor growth and metastasis.^91,339^ PAD inhibitors can significantly decrease the proliferation of cancer cells without affecting the viability of normal cells.^340^ Compounds such as Cl-amidine and F-amidine have demonstrated efficacy in inducing differentiation and apoptosis in various cancer cell lines, including HL60, HT29, TK6, and U2OS.^341^ PAD4 inhibitors have been used clinically to prevent tumor dissemination and treat cancer-associated thrombosis. Effective PAD inhibitors can enhance anti-tumor activity by inhibiting CitH3 in tumors; for instance, in castration-resistant prostate cancer, PAD2-H3Cit26 is considered a novel therapeutic target.^223,342^ Moreover, the combination of PAD inhibitors and HDAC inhibitors is regarded as a strategy for cancer therapy. Compounds such as paclitaxel, minocycline, and streptomycin have been identified as PAD inhibitors; however, due to their poor binding efficacy, they exhibit relatively weak and suboptimal therapeutic effects.^343^ Newer compounds such as O-F-amidine and O-Cl-amidine have shown greater therapeutic efficacy, selectivity, and bioavailability. O-F-amidine, in particular, is markedly more effective than its predecessors, demonstrating an activity 65 times stronger than that of F-amidine and exhibiting a greater preference for PAD1 inhibition.^344^ Recent developments include YW3-56, which not only inhibits PAD4 but also modulates key signaling pathways such as mTORC1, and can obstruct the autophagy of cancer cells, thereby inhibiting their growth.^345^ Continued pharmacological innovation is needed to develop PAD inhibitors with improved selectivity, efficacy, and fewer side effects. This includes targeting specific PAD enzymes that are predominantly expressed in malignant cells without affecting normal tissues.

Histone cit has been identified as a biomarker and therapeutic target in cancer, particularly as PAD4-mediated cit of histone H3 is associated with poor clinical outcomes and a high rate of short-term mortality in patients with advanced cancer, and it can also predict the risk of venous thromboembolism.^231,233,346^ Studies have also indicated that PAD2-mediated cit of histone H3 at arginine 26 promotes malignant progression in multiple myeloma and prostate cancer.^223,347^ Targeting both PAD4 and HDAC2 concurrently emerges as a promising approach in osteosarcoma therapy. This strategy capitalizes on the regulatory capability of histone citrullination to modulate the expression of the tumor suppressor gene OKL38. Such a dual inhibition mechanism suggests a significant potential for therapeutic intervention in osteosarcoma, highlighting a nuanced understanding of histone modifications in cancer treatment.^91,348^ Furthermore, PAD4 inhibitors are utilized to prevent tumor metastasis and thrombosis associated with insulinomas and breast cancer,^349^ and to inhibit hematogenous metastasis of gastric cancer by targeting citrullination-mediated NETs with herbal compounds.^350^ These findings underscore the significance of developing PAD inhibitors that target specific cit sites for cancer treatment and offer new directions for future cancer diagnostics and therapeutics.

The PAD4 protein has emerged as a promising therapeutic target for cancer, offering specific targeting capabilities and a favorable in vivo safety profile against tumor cells. Recognized as an antitumor agent, phenylboronic acid (PBA) can target both primary and metastatic tumors. In this context, researchers have endeavored to enhance PAD4 protein inhibitors by incorporating various PBA components, culminating in the development of highly targeted PAD4 inhibitors. Various experiments have revealed that the m-PBA-modified PAD4 inhibitor, labeled as 5i, exhibited pronounced antitumor activity. Importantly, compound 5i did not act by directly destroying cancer cells; instead, it played a substantial role in curbing the spread of tumor cells. Moreover, the compound named ta3 was found to reduce H3cit in the cell nucleus. The study determined that inhibitors of PBA-PAD4 effectively hinder both the proliferation and spread of breast cancer cells and significantly diminish the development of NETs within the cancerous tissue. This inhibitory action stems from the specific targeting of the PAD4 protein in the nuclei of neutrophils. Furthermore, the PBA-PAD4 inhibitor demonstrated remarkable antitumor activity, suggesting a novel approach for designing efficacious PAD4 inhibitors.^351^

Evodia alkaloid effectively inhibits the la of histones and the expression of HIF1A in PCa cells, thereby obstructing the process of lactate-induced angiogenesis. Simultaneously, it enhances the transcription of Sema3A and suppresses the transcription of PD-L1, collaboratively impeding the formation of blood vessels in PCa and tumor growth.^352^ Wang and colleagues demonstrated that the concurrent use of the PADI inhibitor Cl-Amidine and the AR signaling inhibitor enzalutamide leads to a combined effect, significantly suppressing the proliferation of CRPC cells in vitro and diminishing tumor growth in vivo models. Results from various studies highlight the importance of PADI2 in regulating AR during the advancement of prostate cancer, especially in CRPC, pointing to PADI as a promising therapeutic target for this condition.^223^ I-BET762, I-BET726, and CPI-203 influence histone cr through the modulation of BRD4 levels, subsequently leading to the suppression of growth, movement and invasive behavior in PCa cell lines.^225^

It has been demonstrated in studies that neutrophils can induce the cit of histone H3, leading to NET formation in both mouse and human multiple myeloma cells. One research has confirmed that targeting PAD4 with a novel and specific small molecule inhibitor, BMS-P5, can delay the onset of symptoms in mice with multiple myeloma and significantly inhibit tumor progression.^353^

In studies of multiple sclerosis, abnormal elevation of PAD activity has been observed. To inhibit PAD, researchers developed a non-covalent inhibitor based on an α-amino acid and ethyl isocyanate core structure, where compound 23, containing an imidazole heterocycle, exhibited high selectivity and significant potency against PAD2. In animal models, compound 23 effectively reversed the physical disability induced by experimental autoimmune encephalomyelitis and reduced T-cell infiltration in the brain. This suggests that compound 23 and its analogs hold promise for further development as potential therapeutics for the treatment of multiple sclerosis.^354^

Research indicates the roles of NETs and PAD in a model of endotoxin shock. The study utilized the PAD inhibitor YW3-56, which was found to effectively prolong the survival of mice induced with lipopolysaccharide. The efficacy of the therapeutic intervention on NETs, pro-inflammatory cytokines (including IL-6, TNFα, and IL-1β), and pulmonary damage was evaluated through ELISA and immunostaining methods. Findings from the study revealed that YW3-56 attenuated the inflammatory injury instigated by LPS. This attenuation was marked by the suppression of NET formation, a decrease in inflammatory cytokine levels, and a reduction in lung tissue injury, indicating its potential as a therapeutic agent. This suggests that inhibiting NET formation may serve as a novel strategy in treating endotoxin shock and related inflammation.^355^

#### HDAC inhibitors

HDAC inhibitors are classified into four primary structural groups: hydroxamic acids, cyclic peptides, short-chain fatty acids, and benzamides. This categorization is predicated on the distinctive chemical structures that dictate the inhibitors’ interaction with HDAC enzymes, guiding their use and specificity in epigenetic therapies.^356,357^ The impact of these HDAC inhibitors on HDACs and their encoding genes has been investigated in animal models of MDD, indicating their antidepressant effects. Trichostatin A (TSA) is capable of reversing hippocampal transcriptome alterations in rats induced by maternal care during early life.^358^ Vorinostat, recognized in the scientific community as SAHA, holds the distinction of being the inaugural HDAC inhibitor sanctioned by the U.S. Food and Drug Administration for clinical application. Research into Vorinostat has unveiled its capability to ameliorate behaviors associated with MDD and to restore the expression of Glial Cell Line-Derived Neurotrophic Factor (GDNF) in mice subjected to Chronic Unpredictable Mild Stress, underscoring its potential for therapeutic intervention in neuropsychiatric disorders.^359,360^ Valproic acid (VPA) has been found to influence the expression of BDNF, GSK-3β, CORT, and MC4R, and exhibits antidepressant properties.^361–363^ MS-275, as a selective inhibitor targeting Class I HDACs, affects the expression of CREB, BDNF, CORT, RAC1 and GJA5.^364^ Although HDAC inhibitors have demonstrated antidepressant properties in animal models, there remain limitations to be addressed before their widespread clinical application, including the potential inhibitory effects on the ac of non-histone proteins such as alpha-tubulin, HIF-1 alpha, Stat3, and beta-catenin.^365–368^ For instance, Farydak, an HDAC inhibitor approved by the FDA for the treatment of multiple myeloma, has exhibited severe side effects such as gastrointestinal toxicity, thrombocytopenia, bone marrow suppression, fatal cardiac ischemic events, arrhythmias, electrocardiogram alterations, local and systemic infections, as well as hepatic dysfunction.^369^ Studies have shown that combining HDAC inhibitors with common antidepressants such as fluoxetine can significantly reduce MDD-related behaviors, suggesting that HDAC inhibitors may have potential for use in conjunction with conventional antidepressants in the future treatment of MDD. Lithium has been found to decrease the expression of HDAC1, 3, 4, 5, 7, 8, and 10.^370,371^ Olanzapine enhanced the ac of histone H3 at the promoter regions of BDNF in the hippocampal area of rats with MDD, while concurrently inhibiting HDAC5.^372^ Combined treatment with lithium and valproic acid has been shown to induce BDNF expression and exhibit neuroprotective effects in MDD.^373,374^

Effective compounds found in Xiao Yao San, such as quercetin, rutin, saikosaponin D, ferulic acid, and curcumin, have been identified through high-performance liquid chromatography (HPLC).^375^ These compounds have shown potential in modulating histone modifications and treating depression, as seen in quercetin’s ability to regulate HDAC and HAT activities, ameliorate cognitive deficits, and enhance the expression of neural plasticity markers.^376^ The realm of histone modification extends beyond the treatment of MDD and is being explored as a therapeutic target for an array of neurological conditions such as PD, AD, HD, and SMA. For instance, HDAC inhibitors like TSA, SAHA, VPA, and sodium butyrate have demonstrated efficacy in rodent models of Parkinson’s Disease. These inhibitors elevate the expression of neurotrophic factors, including GDNF and BDNF, safeguard dopaminergic neurons, and enhance dopamine synthesis, thereby manifesting their potential in neuroprotective strategies.^377–380^ The neurotoxicity of α-synuclein can also be mitigated by HDAC inhibitors, thereby ameliorating the symptoms of Parkinson’s Disease.^381^ Levodopa, a medication commonly used for PD, has been found to reduce the ac of histone H4.^382^ VPA can inhibit beta-amyloid, a peptide highly associated with AD.^383,384^ HDAC inhibitors such as tubastatin A and ACY-1215 have been found to reduce the hyperphosphorylation of tau protein in AD.^385^ Donepezil, a common medication used in the treatment of AD, has been discovered to inhibit the binding between HDAC6 and the BDNF promoter in the cortex, thereby resulting in the overexpression of BDNF.^386^ HDAC inhibitors, including TSA, SAHA, sodium butyrate, RGFP966, and LBH589, have been shown to ameliorate symptoms in mouse models of HD.^387–390^ In models of Spinal SMA, a variety of HDAC inhibitors have been found to induce the expression of the survival motor neuron 1 gene, which is crucial for the disease.^391–396^

Although HDAC inhibitors have demonstrated neuroprotective effects in animal models, their clinical application still faces challenges, such as their potential for severe side effects and the ability to penetrate the blood-brain barrier. HDAC inhibitors have been approved by the United States FDA for clinical trials in cancer treatment, but human clinical trials for depression have not yet been conducted.

Therapeutic strategies for lung cancer are closely linked with changes in histone la and cr. This finding suggests a potential new approach to improve PEM sensitivity. Proteomic studies of histones have uncovered the pivotal role of the HDAC1 in deacetylation complex in the deacetylation of lysine cr at the H3K18cr site on histone 3. One research has notably observed that HDAC1 inhibition diminishes both the presence of H3K18cr histone and RNA polymerase II at the caspase-1 promoter in cellular environments. Moreover, reducing HDAC1 levels is found to significantly decrease the growth rate of NSCLC cells resistant to PEM. These insights suggest that HDAC1, when crotonylated and associated with caspase-1, could play a role in influencing PEM resistance by specifically acting on GSDMD.^397^

Glioma histone deacetylase inhibitors are recognized for their anti-tumor properties. These elements have been noted to initiate multiple types of histone post-translational modifications in glioma cells, notably including acetylation and bu. This suggests a profound connection between their antitumor efficacy and the processes of histone acetylation and bu. Assessing the levels of histone bu and pr in cancer cells can provide indirect insights into the pharmacological actions of HDAC inhibitors.^398^

Studies have indicated that the HDAC inhibitor, SAHA, originally used for treating cutaneous T-cell lymphoma, also shows substantial effectiveness against neuroblastoma. Through detailed quantitative proteomic assessments, a significant induction of histone bu and acetylation was observed following SAHA treatment. This highlights the crucial role of HPTMs in its anti-tumor activity and encourages the exploration of novel anti-tumor drugs that target histone bu as a primary mechanism.^399^

#### Others

The findings of the study suggest that the primary mechanism of the anti-cancer properties of various drugs lies in their ability to target histone succ levels and the enzymes associated with the succ process. For example, the ability of aspirin to limit tumor proliferation is associated with its capacity to reduce the succ level of PGAM1. This action effectively impedes the proliferation of liver cancer cells and disrupts the glycolytic pathway.^188^ One research indicates that suppressing the expression of succinyltransferases, such as KAT2A, reduces tumor cell proliferation and overall tumor growth.^126^ This suggests a promising approach for utilizing histone succ in tumor therapies. Furthermore, it has been observed that aspirin inhibits glycolysis and increases the efficacy in cancer cells by reducing the total bib of ENO1.^400^

Resistance to temozolomide (TMZ) remains a significant obstacle in the treatment of GBM.^401^ The clinically approved antiepileptic drug stiripentol can cross the blood-brain barrier and inhibit the activity of lactate dehydrogenase A/B (LDHA/B), acting as a la inhibitor and rendering GBM cells more sensitive to TMZ both in vitro and in vivo.^179^ Furthermore, an increase in mitochondrial reactive oxygen species (mROS) and glycolysis has been identified in pulmonary hypertension.^402^ Investigations have uncovered that hypoxia-driven mitochondrial mROS hinder the hydroxylation process of HIF-1α. This obstruction fosters a glycolytic shift within Pulmonary Artery Smooth Muscle Cells (PASMCs) by the activated HIF-1α/PDK1&PDK2/phosphorylated-PDH-E1α pathway, leading to an escalated build-up of lactate and histone la. Such an increase in histone la at loci of HIF-1α target genes, which include Bmp5, Trpc5, and Kit, is linked to the promotion of PASMC proliferation. Diminishing Pdk1&2 levels tempers lactate concentration, histone la markers, and the proliferation of PASMCs. Additionally, pharmacological intervention with lactate dehydrogenase inhibitors has been shown to curtail histone la and mitigate PASMC proliferation and vascular remodeling in rats with hypoxic Pulmonary Hypertension.^403^ The combined use of histone la and macroautophagy/autophagy inhibitors with bevacizumab therapy has demonstrated significant therapeutic efficacy in preclinical models of patients resistant to bevacizumab.^404^

### Inhibitors indirectly interfering with the process of novel HPTMs

Research indicates that the expression of hexokinase 2 (HK2) plays a pivotal role in activating hepatic stellate cells through lactylation-mediated histone regulation of gene expression. Targeting HK2 can inhibit the activation of hematopoietic stem cells and reduce liver fibrosis systemically, showcasing the potential of HK2 as an effective therapeutic target for liver fibrosis.^405^

Research reveals that lactate influences the treatment and prognosis of CRC by inhibiting RARγ, primarily by remodeling the functionality of macrophages within the tumor microenvironment. Additionally, the study identifies nordihydroguaiaretic acid (NDGA) as an effective therapeutic agent that directly binds to RARγ and inhibits the TRAF6-IL-6-STAT3 signaling pathway, offering a new therapeutic strategy targeting pro-tumoral macrophages in CRC.^406^ In another study, researchers conducted a global mapping of HPTMs in CRC cells treated with largazole-7. They noted that the drug’s selectivity for cancer cells was more than 100 times greater than that for normal cells. Changes in lysine methylation and bu were observed at 68 core histone sites following drug exposure, suggesting that largazole-7 could counteract lysine bu.^407^ This finding suggests that the anti-tumor efficacy and high specificity of largazole-7 may be attributed to its targeted action against histone bu in CRC cells. Additionally, it has been demonstrated that the cancer treatment delivery ligand, GnRH-III, shows enhanced tumor-inhibitory effects and higher binding affinity when combined with bu.^408,409^ Moreover, precise rectification of irregular histone la markedly restrains both the development and spread of ccRCC in live models. Even more crucial is the finding that simultaneously targeting histone la and PDGFRβ greatly amplifies the treatment effectiveness. This research underscores the vital importance of HPTMs, particularly histone la, in the advancement of ccRCC, indicating that interrupting the reinforcing cycle between histone la and PDGFRβ signaling may provide an innovative approach to treating ccRCC patients.^209^

Pan et al. identified a unique triterpene anti-tumor compound, DML and demonstrated its efficacy in inhibiting HCC progression by targeting two tumor-promoting HPTMs sites: H3K9la and H3K56la. The in vivo mechanism of DML in regulating H3 la was confirmed through a tumor xenograft model in nude mice, highlighting DML’s potential as a promising candidate for HCC treatment.^410^ Additionally, A link between la of H3 histone and the anticancer properties of RJA has been demonstrated in studies. Their research revealed that RJA impedes the progression of HCC by disrupting lactate generation and blocking la at the histone sites H3K9la and H3K14la.^411^

It has been discovered that glioblastoma stem cells contribute to the accumulation of crotonyl-CoA and the cr of histone H4 lysine by regulating the mechanism of lysine catabolism. Furthermore, experimental evidence has shown that inhibiting histone lysine cr can effectively suppress tumor progression.^412^

Andrographolide (AGP) can significantly reduce aortic valve calcification by inhibiting the p300 enzyme’s la of histones, particularly at the H3K9 and H3K18 sites. This la is associated with the expression of Runx2, a key factor influencing bone metabolism and calcification. By inhibiting these la sites, AGP diminishes Runx2 expression, thereby mitigating calcification.^413^

## Common techniques and recent advances in Hptms research

**Fig. 9:**
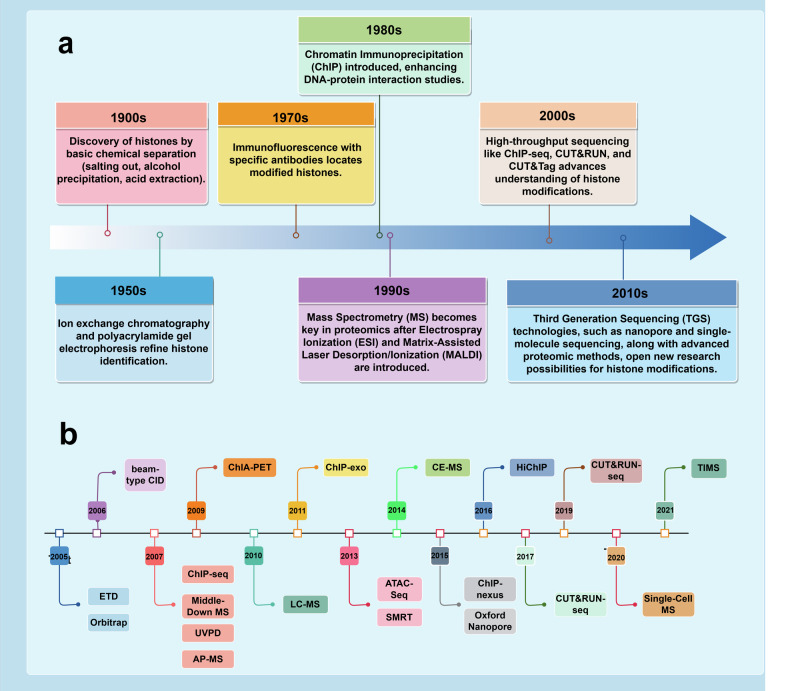
Timeline of HPTMs research techniques. **a** The progression in researchers’ methods for studying histones from the early 20th century to the early 21st century. **b** The specific advancements and technical developments in studying histones based on NGS, TGS, and MS over the past 15 years

In the domain of epigenetics research, a plethora of sophisticated technologies have been developed to study the epigenomic states of genomes and their underlying molecular mechanisms. ChIP-seq is utilized to investigate protein-DNA interactions and histone modifications across entire genomes.^414,415^ DNase-seq and ATAC-seq assess chromatin accessibility, with the latter being particularly suited for samples with a low cell count.^416,417^ FAIRE-Seq^418^ and MNase-seq^419^ are employed to identify open chromatin regions and map nucleosome positions. High-throughput sequencing strategies such as BS-Seq,^420^ oxBS-Seq,^421^ fCAB-Seq,^422^ and CAB-Seq^423^ have been formulated to explore DNA and RNA modifications. Novel techniques like CUT&Tag and CUT&RUN leverage antibodies to provide high-resolution and cost-effective solutions for analyzing transcription factors, histone modifications, and protein-DNA interactions.^424–426^ Each technique comes with its own set of strengths and limitations, and often a combination of multiple methods is required. With growing demands, it becomes especially critical to develop more streamlined and practical technologies for the future **(**Fig. 9**)**.

### Next-generation sequencing technology (NGS)

NGS technologies has greatly expanded the methodologies available for protein research, particularly through the integration of ChIP with sequencing known as ChIP-Seq, enabling scientists to comprehensively map epigenetic marks across the entire genome.^427,428^ This is particularly significant for the prognosis of diseases and the development of therapies. Since its first application by Barski and colleagues in 2007, ChIP-Seq has become a pivotal tool for studying epigenetic modifications due to its cost-effectiveness, efficiency, high sensitivity, and extensive genomic coverage.^429^

In 2017, the Henikoff laboratory introduced CUT&RUN, a technique that addresses issues of high false-positive rates and poor antibody specificity seen in traditional ChIP-seq. Its advantages include applicability to small cell samples, high signal-to-noise ratio, low cell requirements, and the ability to detect distal transcription factor binding within three-dimensional space.^426,430^ The CUT&Tag technology, introduced in 2019, precisely identifies and characterizes characteristic peaks of histone modifications through methods such as GoPeaks, suitable for genome-wide analysis, and can be combined with single-cell library construction techniques for high-resolution measurements, yielding high-quality single-cell chromatin modification data.^425,431^ The CUT&Tag-BS method enables simultaneous detection of histone modifications and DNA methylation, particularly fitting for limited sample volumes.^432^

Advancements in single-cell sequencing technologies have led to the development of ACT-seq, which allows for the analysis of histone tail modifications in minute quantities, including single cells, and enables the construction of thousands of single-cell libraries within a single day.^433^ Moreover, Yang and colleagues have developed the scChIX-seq technique, which combines experimental and computational methods to concurrently analyze multiple histone marks within individual cells. This approach supports multimodal analysis of antibody-mediated chromatin studies, endowing scChIX-seq with extensive potential applications in epigenetic research^434^ (Table 3).

**Table 3:** Comparison of common histone modification analysis techniques

Parameter	ChIP-seq	CUT&RUN	CUT&Tag	MiniON	Oxford Nanopore Technologies (TGS)	ACT-seq technique	Mass spectrometry (MS)
Starting Material	1 M cells	50k cells	50k-1k cells	Varies	Varies	Low input	Varies
Library Construction	End-repair adapter ligation	End-repair adapter ligation	PCR to add adapters	N/A	N/A	N/A	Sample preparation varies
Sequencing Depth	20 M reads	8 M reads	2 M reads	High	High	High sensitivity	Depth varies
Protocol Duration	Approx. 1 week	1-2 days	1-2 days	Varies	Varies	1 day	1-3 days
Advantages	Widely validated,^427–429^ suitable for a variety of protein targets 1. Reflects target proteins on DNA: Accurately shows proteins bound to DNA sequences. 2. Standard genome-wide technique: Ideal for studying DNA-Protein Interactions (DPI) at a genome-wide level.	Low sample requirement, simple operation, low background noise, high signal-to-noise ratio.^426,430^ 1. Signal-to-noise ratio significantly higher than traditional ChIP-seq. 2. Generates chromatin fragments with restriction digestion. 3. Capable of detecting long-distance 3D binding sites of transcription factors. Requires a small number of cells.	Extremely low cell requirement, simplified procedure, low cost, high signal-to-noise ratio. 1. Superior signal-to-noise ratio and lower background. 2. Higher reproducibility. 3. More efficient in recognizing chromatin characteristics. 4. Capable of analyzing extremely small cell quantities, including single cells. 5. Shorter experimental cycle.^425,431^	Long-read sequencing capability	Long-read capability, real-time sequencing, suitable for complex genetic structures	High sensitivity for single-cell transcription analysis	High precision in protein identification
Disadvantages	Requires large number of cells, complex operation, high background noise 1. Requires many cells: High cell quantity needed for standard experiments. 2. Poor reproducibility: Variability in experimental results. 3. Low signal-to-noise ratio: Background noise affects data clarity.	Lower technical challenge but not suitable for some targets. 1. CUT&RUN requires reagent standardization, the published pA/MNase purification protocols are complex. 2. The original protocol is highly sensitive to digestion time.	High technical challenge, not suitable for all targets. 1. The non-crosslinking protocol of CUT&Tag is not always suitable. 2. CUT&Tag may introduce biases.	Higher cost per base, higher base calling error rate	Limited continuity, less accurate	Technique still under development	Requires expensive equipment
Sensitivity and Specificity	High sensitivity, suitable for detecting low-abundance marks	High specificity, suitable for rapid detection of specific protein-DNA interactions	High sensitivity, high specificity, suitable for ultra-low cell numbers	Suitable for complex genome structures	High sensitivity, long-read technology	Extremely high sensitivity and specificity	Extremely high precision for quantitative analysis
Sample Compatibility	Fixed samples, frozen tissues	Fixed and non-fixed samples	Fixed and non-fixed samples	Broad sample compatibility	Broad sample compatibility	Low input, single cells	Broad range of sample types
Data Processing Complexity	High	Medium	Medium	Medium	High	Medium	High
Cost	Moderate	Low	Low	High	High	Moderate	High
Technology Maturity	Mature	Emerging	Recently developed	Emerging	Mature	Developing	Mature
Technical Limitations	Sensitive to environmental conditions, requires large samples	Some targets not suitable, lower technical maturity	High technical demands may limit widespread use	High cost, high error rates	High error rates, limited continuity	Still under development, limited application scope	Requires expensive equipment, complex operation
Applicability	Widely used in epigenetics research	Suitable for precision genomics with low cell numbers	Especially suitable for studies requiring high sensitivity and specificity	Suitable for complex genomic analysis and long-read sequencing needs	Suitable for in-depth research into histone modifications and other complex genetic analyses	Ideal for single-cell histone modification profiling	Ideal for quantitative protein analysis in complex biological samples
Reference	^414^^,415,427–429^	^426,430^	^425,431,432^	^442^	^438^	^434^	^445,446^

#### Third-generation sequencing (TGS)

TGS, also known as single-molecule sequencing, operates by allowing a single DNA strand to pass through a nanopore, with the sequence of bases identified using fluorescence or electrical blockade. TGS addresses some limitations of second-generation sequencing, particularly overcoming issues of limited read length in NGS, by enabling continuous sequencing of hundreds of thousands of bases. TGS is versatile, applicable not only to DNA and RNA sequencing but also to direct observation of epigenetic patterns on proteins and genetic material.^435^ Furthermore, TGS offers distinct advantages over NGS in handling complex DNA scenarios, thus facilitating more in-depth research into histone epigenetic modifications.^436^ For instance, TGS has been identified as a unique tool for investigating HPV sequences.^437^

In 2014, Oxford Nanopore Technologies introduced the MiniON, the first commercially available nanopore sequencer.^438^ Despite challenges like limited continuity and higher error rates in base calling, nanopore technology’s potential has been widely recognized.^439^ The main TGS platforms are Pacific Biosciences and Oxford Nanopore Technologies, each offering unique solutions. Nanopore technology pairs each nanopore with a nucleic acid cleavage enzyme, whereas Pacific Biosciences achieves sequencing by introducing fluorescently labeled bases alongside the target sequence, using DNA polymerase. The introduction of these platforms has significantly propelled the advancement of TGS technology.^440,441^

Moreover, novel methodologies based on nanopore technology such as nanoHiMe-seq^442^ and SMOOTH-seq^443^ have been developed, which can be utilized to explore the intricate interactions of epigenetic modifications within the genome or to identify structural variants and extrachromosomal circular DNA within single cells. The base accuracy rate of Third-Generation Sequencing is currently around 90%, and it also confronts the challenge of extracting large macromolecules, high-molecular-weight DNA, and intact RNA from clinical samples.^444^

#### Mass spectrometry (MS)

MS is a precise analytical tool that measures the mass-to-charge ratio (m/z) of ionized molecules to accurately determine their molecular weight.^445,446^ MS has emerged as the method of choice for the identification and quantification of HPTMs due to its objectivity, comprehensiveness, and precise quantification.^447^ Compared to traditional antibody-based methods, mass spectrometry boasts the advantage of detecting any type of HPTM in a single experiment without prior knowledge of the modification type or location. Additionally, mass spectrometry can accurately quantify HPTMs, addressing issues such as cross-reactivity and epitope masking associated with antibody methods.^448^

Liquid chromatography-tandem mass spectrometry (LC-MS/MS) has become an integral advancement in MS-based targeted proteomics, offering high sensitivity and precision in protein analysis, and excelling particularly in the large-scale quantitative analysis of proteins and their post-translational modifications. These technologies play a crucial role in identifying and validating cancer biomarkers, facilitating early diagnosis, unraveling molecular mechanisms, and guiding therapeutic strategies.^449–451^ Through LC/MS, researchers can analyze histone HPTM patterns in normal and tumor tissues, such as the observed decrease in H3K27me3 and an increase in H3K9me and H3K36me1/me2 in aggressive triple-negative breast cancer.^452,453^

Despite its widespread application, LC/MS’s limitation lies in the loss of spatial information. In contrast, Matrix-Assisted Laser Desorption/Ionization Mass Spectrometry Imaging (MALDI-MS Imaging) showcases unique advantages in the analysis of histone variants and HPTMs, especially in providing spatial information.^454^ By directly applying MALDI matrix onto tissue samples and using laser scanning, this technique generates a mass spectrum for each measurement point, offering spatial distribution information akin to immunohistochemistry while simultaneously analyzing multiple peptides. MALDI Imaging has been successfully applied to identify regulated histone HPTMs/variants in various disease states, such as the increase of H4K16 acetylation and K20 dimethylation in hepatocellular carcinoma.^455,456^ To overcome challenges associated with MALDI Imaging, such as analysis resolution and protein quantification issues, this technique is often coupled with LC/MS, providing new avenues for histone-based disease research and the identification of epigenetic markers.

While bottom-up mass spectrometry methods are popular for their high throughput and efficiency, they often sacrifice information pertaining to combinatorial HPTMs patterns and variant differences. In contrast, middle-down and top-down approaches can provide richer information but face challenges in the clinical application such as data analysis complexity and interpretation. These methods require specific instrument setups, are analytically complex, and demand larger amounts of starting material, which limits their widespread application in clinical settings.^456^ Recently introduced direct infusion mass spectrometry techniques, which forgo traditional HPLC separation, can analyze 200 HPTMs within one minute, addressing reproducibility issues associated with nanoHPLC separations and demonstrating the potential to process up to 1000 samples per day.^457^ This offers hope for their application in clinical samples, though the suitability of these technologies will require further validation. However, their integration with targeted MRM acquisition workflows promises to be an ideal choice for clinical sample processing.^458^

Additionally, an array of pre-mass spectrometry techniques such as Trapped Ion Mobility Spectrometry (TIMS),^459^ Ion Mobility Spectrometry (IMS),^460^ and various fragmentation methods (such as CID, beam-type CID, ETD, and UVPD^461^) have significantly advanced the study of HPTMs. These technologies, by enhancing the selectivity and sensitivity of analysis, have bolstered the capacity to identify and quantify protein modifications in complex biological samples. Advances in high-resolution mass spectrometry technologies, such as Orbitrap^462^ and FTICR,^463^ are crucial for identifying subtle mass differences in molecules, especially in the study of HPTMs. These technologies provide precise mass measurements, aiding in a deeper understanding of the roles proteins play in cellular functions and disease progression.

#### Others

BioID technology is an effective and widely used method for proximity-dependent labeling of proteins in eukaryotic cells. This technique is designed to screen for interacting and proximal proteins within their natural cellular environment, and it has also been utilized in histone research.^464^ Additionally, technologies like BioID can complement traditional affinity purification mass spectrometry (AP-MS) methods.^465^ Recently, the development of in vitro BioID (ivBioID) has enabled scientists to depict the microenvironment of the histone H3 variant CENP-A with higher temporal and spatial resolution.^466^ The future joint application of ivBioID and MS is also worth looking forward to. This advancement provides new perspectives and tools for studying protein interactions and cellular functions.

## Conclusion and perspective

In summary, this review encapsulates the historical progress of histone modifications, their basic structures, enzymes involved, and biological functions, while also emphasizing the significant contributions of nine novel histone modifications in advancing the treatment of various diseases, particularly cancer. The review emphasizes the impact of novel histone modifications on diseases treatment, particularly through metabolic pathways, their potential in overcoming drug resistance, and their synergistic effects with other treatments. Furthermore, we also discuss the importance of advanced techniques in histone research, which are crucial for comprehending the cancer physiology. The summary of the relationship between histone modifications and diseases has enriched our understanding of diseases and led to the development of targeted inhibitors. These advancements not only hold promise for refining current diseases therapeutic strategies but also open avenues for more personalized and effective approaches to diseases management in the foreseeable future.

Although numerous researches have highlighted the importance and therapeutic potential of HPTMs in various diseases, the specific roles and mechanisms of HPTMs in the development and treatment of cancers remain largely unclear. Furthermore, the landscape of epigenetic regulation becomes even more complex when considering competitive or antagonistic interactions that may arise as different modification pathways converge on the same amino acid residue.^4^ For instance, there is a balance between the ac and ma of histone H3 at lysine 9,^467^ as well as a dynamic competition between ac and bu at lysines 5 and 8 of histone H4.^468^ Additionally, within the field of epigenetics in cancer therapy, the roles of “writers”, “erasers” and “readers” present challenges, especially in terms of their specificity to individual HPTMs. These proteins exhibit affinity for a broad range of HPTMs, which could lead to a variety of side effects. The interaction between histone modifications and other forms of epigenetic alterations, like DNA ma, requires additional exploration.^469^

Over the past few decades, significant progress has been made in the research of various histone inhibitors. However, the research remains unbalanced, and the clinical potential has not been fully exploited. Developing highly efficient, selective inhibitors of histone modifications is a vital need for future research. Dual inhibitors, such as tyrosine kinase and HDAC inhibitors,^470,471^ VEGFR-2/HDAC inhibitors,^472^ and FGFR/HDAC inhibitors,^473,474^ hold immense application potential in cancer therapy. Moreover, several novel dual inhibitors have entered clinical trial phases, demonstrating feasibility and efficacy in the treatment of solid tumors.^475^ The roles of novel HPTMs in other diseases remain worthy of investigation. For instance, emerging HPTMs continue to have a distinctive impact on processes like embryonic development; however, research into the connection between histone modifications and their developmental implications or therapeutic studies remains scant. Moreover, many traditional HPTMs are crucial regulatory factors of cellular functions. The interplay or competitive mechanisms between them and novel HPTMs and their roles in diseases require further investigation. In addition, research on the “readers” of various novel HPTMs remains a blank slate. Given the ubiquity of various acylation modifications, there must exist more types and functions of “writers,” “erasers,” and “readers” in novel HPTMs, represented by la, thus warranting further exploration. This will provide new ideas and targets for improving cancer treatment outcomes and tumor prognosis. There is an anticipation that more research in the future will delve into these diseases, providing a deeper understanding of these epigenetic influences. With the continuous advancement of novel analytical techniques, it is anticipated that more types of histone modifications and new target inhibitors will be discovered, as well as hybrids that not only possess multi-target effects but also significantly enhance therapeutic efficacy in vivo. This holds tremendous potential for the future treatment of various diseases, particularly cancer.
